# Multiple cis‐regulatory elements collaborate to control *mdka* expression in telencephalic neural stem cells of adult zebrafish during constitutive and regenerative neurogenesis

**DOI:** 10.1111/febs.70345

**Published:** 2025-11-19

**Authors:** Jincan Chen, Masanari Takamiya, Agnes Hendriks, Tanja Beil, Csilla Várnai, Nicolas Diotel, Sepand Rastegar

**Affiliations:** ^1^ Institute of Biological and Chemical Systems‐Biological Information Processing (IBCS‐BIP) Karlsruhe Institute of Technology (KIT) Germany; ^2^ Department of Cancer and Genomics Sciences University of Birmingham UK; ^3^ Université de La Réunion, INSERM, UMR 1188, Diabète athérothrombose Thérapies Réunion Océan Indien (DéTROI) Saint‐Pierre France

**Keywords:** Cis‐regulatory elements, gene transcription regulation, *mdka*, neurogenesis, radial glial cells, regeneration, zebrafish

## Abstract

Zebrafish is a powerful animal model for studying nervous system regeneration due to its remarkable regenerative abilities and the availability of diverse molecular tools. After telencephalic brain injury, neural stem cells (NSCs) in the ventricular zone (VZ) become activated, proliferate, and generate new neurons essential for brain repair. However, the molecular mechanisms regulating these processes remain unclear. Here, we investigate the transcriptional regulation of *midkine‐a* (*mdka*), a heparin‐binding growth factor gene encoding the secreted protein Midkine‐a (Mdka), which is upregulated after injury in radial glial cells (RGCs), the *bona fide* NSCs of the adult zebrafish telencephalon. Using genome‐wide bioinformatic analysis, we identified six putative cis‐regulatory elements (CREs) associated with *mdka*. Transgenic assays revealed that these CREs coordinate *mdka* expression during both development and regeneration. In the zebrafish embryo, CRE2, CRE3, CRE4, and CRE6 are required for EGFP expression in the nervous system, with CRE3 showing the strongest activity. In the adult telencephalon, CRE2, CRE4, and CRE6 are active in NSCs, with CRE2 best mimicking *mdka* expression at the ventricular zone. Importantly, individual CREs could not fully reproduce endogenous *mdka* expression, especially under regenerative conditions. In contrast, a combined CRE2346 construct closely recapitulated *mdka* expression in both the embryo and adult telencephalon under homeostatic conditions. These results suggest that *mdka* expression is controlled by a modular and cooperative cis‐regulatory architecture that enables precise gene regulation during development, telencephalon homeostasis, and regeneration.

## Graphical abstract

*midkine‐a (mdka)*, a pan‐injury response gene in vertebrates, is precisely regulated in the zebrafish telencephalon by a modular cis‐regulatory architecture. This control ensures proper expression during development, adult brain homeostasis, and regeneration. Transgenic reporter lines reveal that cis‐regulatory elements 2, 3, 4, and 6 together faithfully replicate endogenous *mdka* expression, highlighting their combinatorial role in orchestrating spatial and temporal gene regulation.

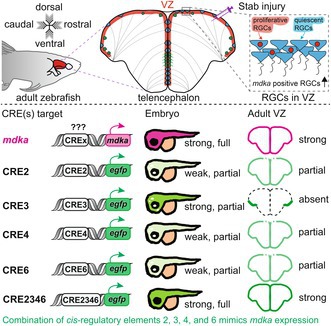

**Abbreviations**
CRE: cis‐regulatory element
Dm: dorsomedial of the telencephalon
dpf: days post‐fertilization
dpl: days post‐lesion
EGFP: enhanced green fluorescent protein
FISH: fluorescent *in situ* hybridization
hpf: hours post‐fertilization
IHC: immunohistochemistry
kb: kilo bases
*mdka*
: *midkine‐a*
mRNA: messenger RNA
NSCs: neural stem cells
PCNA: proliferating cell nuclear antigen
RGCs: radial glial cells
S100β: S100 calcium‐binding protein β
*Tg*
: transgenic line
TSS: transcription start site
VZ: ventricular zone

## Introduction

Understanding the molecular mechanisms underlying tissue and organ regeneration is critical for addressing the growing challenges posed by neurodegenerative diseases, such as dementia, Parkinson's disease, and Alzheimer's disease. These diseases place a significant medical and societal burden on an aging population, underscoring the urgent need for effective therapeutic strategies to address these conditions. Zebrafish has emerged as a key vertebrate model for studying regeneration due to its exceptional ability to regenerate tissues throughout both embryonic and adult stages [1, 2, 3, 4, 5, 6]. Unlike most mammals, zebrafish can regenerate organs like the heart, fin, retina, spinal cord, and specific brain regions without scarring and obvious disabilities, which makes them invaluable for studying regenerative processes [2]. With over 70% genetic similarity to humans [7] and a vast number of mutants [8], transgenic lines [9, 10, 11], and genomic resources such as the DANIO‐CODE Data Co‐Ordination Center (DCC) (https://danio‐code.zfin.org/), zebrafish provide a unique platform to study gene function, regulation, and cellular dynamics during embryonic development, adulthood under homeostatic and regenerative conditions [12, 13, 14]. In zebrafish, the adult telencephalon regenerates by activating dormant RGCs in the ventricular zone (VZ), which produce new neurons to replace those lost from injury [15, 16]. Recent transcriptomic studies have identified several genes significantly upregulated following injury [17, 18, 19, 20], one of the most notable being *midkine‐a* (*mdka*), a heparin‐binding growth factor with multiple roles in development, repair and diseases [21, 22, 23]. Interestingly, *mdka* expression increases in quiescent RGCs after telencephalic injury, resembling the behavior of *id1* (*inhibitor of differentiation 1*) [21], a transcriptional regulator downstream of the BMP signaling pathway [24, 25]. Both *mdka* and *id1* are predominantly expressed in quiescent type 1 cells (RGCs) and are absent in type 2 cells corresponding to actively dividing cells [21]. Both type 1 and type 2 cells express glial markers such as S100β and GFAP, while type 2 cells in addition express the proliferation marker PCNA (proliferating cell nuclear antigen). Lineage tracing experiments have demonstrated that type 2 cells differentiate into type 3 neuroblasts, which migrate and mature into specific neuronal types [26, 27].

Beyond the brain, *mdka* has been implicated in the regeneration of other tissues in zebrafish and other vertebrates, including the heart, fin, and retina, suggesting that it may play a common role in the regenerative process [28, 29, 30, 31, 32, 33, 34, 35]. The zebrafish *midkine* family includes two genes: *mdka* and its paralog *midkine b* (*mdkb*), the result of a gene duplication event [36]. However, only *mdka* is significantly upregulated after injury, highlighting its specific role in regenerative responses [21]. This study investigates the transcriptional regulation of *mdka* in the zebrafish telencephalon, focusing on its regulation in NSCs during neurogenesis and regeneration. We aim to uncover the molecular mechanisms behind its transcription by identifying and characterizing cis‐regulatory elements (CREs) that control *mdka* expression in both homeostatic and regenerative contexts.

Our findings, using genome‐wide bioinformatic approaches and validation by transgenesis, reveal that multiple CREs work together to regulate *mdka* expression. In embryos, CRE2, CRE3, CRE4, and CRE6 are critical for nervous system expression, while in the adult telencephalon, CRE2, CRE4, and CRE6 are essential for expression in NSCs. Notably, none of the CREs alone was sufficient to fully recapitulate *mdka* expression in the embryonic nervous system or adult telencephalon. These findings highlight the complexity of *mdka* regulation, where individual CREs contribute differently to expression patterns depending on molecular and environmental signals. Furthermore, a transgenic EGFP reporter line containing CRE2, CRE3, CRE4, and CRE6 best mimicked endogenous *mdka* expression in both zebrafish embryos and the adult telencephalon under homeostatic and regenerative conditions, emphasizing the role of multiple molecular signals in *mdka* regulation.

## Results

### Identification and characterization of *mdka* CREs in the zebrafish embryo

**Fig. 1:**
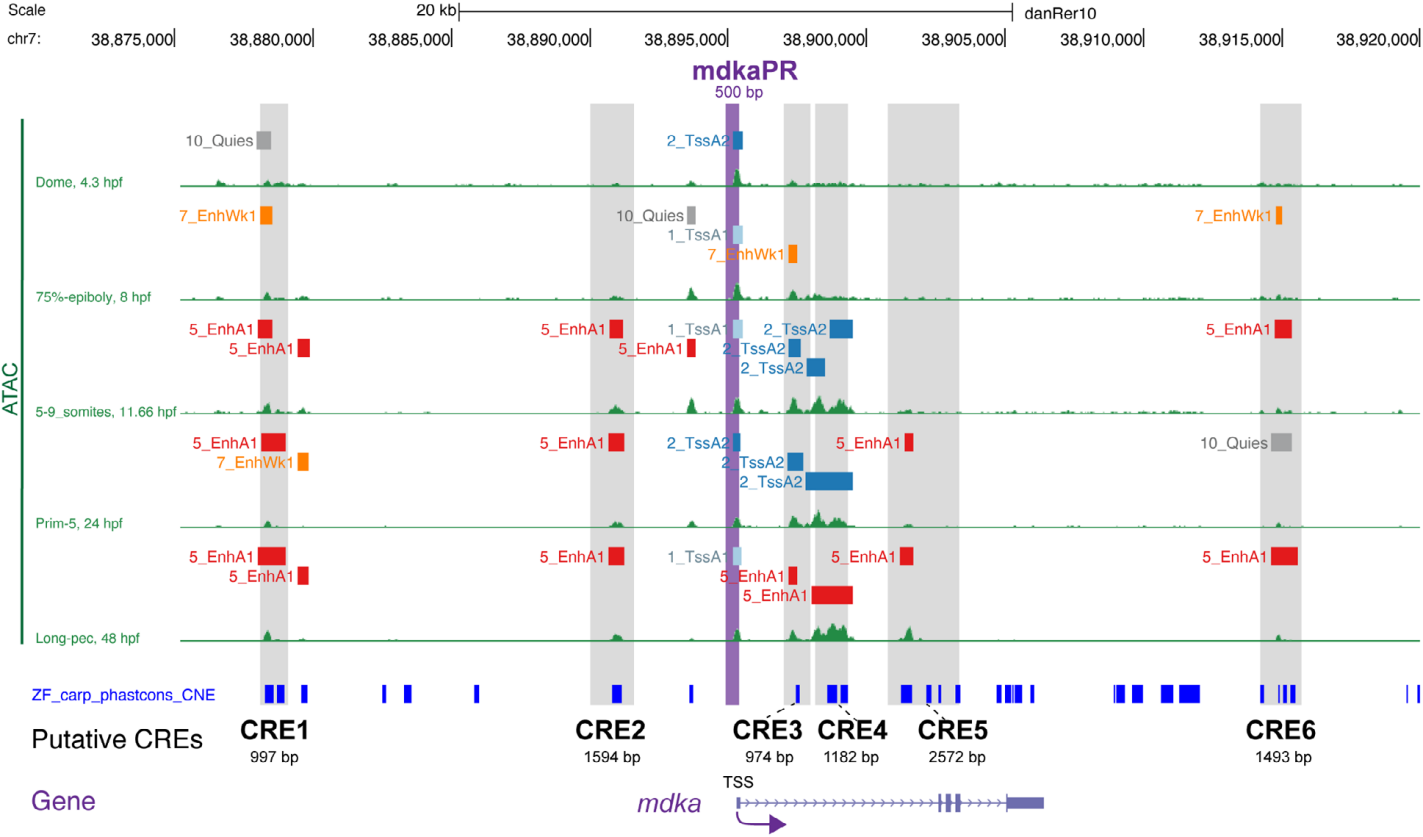
Identification of potential *mdka* regulatory sequences based on embryonic developmental ATAC‐seq data. Snapshot of UCSC genome browser tracks displaying ATAC‐seq data from different embryonic developmental stages (Dome, 75%‐epiboly, 5–9 somites, Prime‐5 and Long‐pec) across a 45‐kilobase region (chr7: 38875000–38920000; danRer10 assembly). Green peak profiles represent open chromatin regions identified by ATAC‐seq. Color‐coded horizontal bars indicate the genomic position of putative regulatory elements based on PADRES (Predicted ATAC‐seq‐supported developmental regulatory elements): promoters (1_TssA and 2_TssA2, blue), enhancers (5_EnhA1, red; 7_EnhWk1, orange), and other elements (e.g., 10_Quies, gray). The gray box highlights six putative *mdka* CREs (CRE1 to CRE6) identified in this study, while the magenta box marks the putative *mdka* promoter region. The relative positions of all CREs are shown in reference to the *mdka* gene and its transcription start site (TSS). PhastCons conservation scores derived from zebrafish‐carp genome alignment highlight conserved non‐coding regions (CNEs) associated with the *mdka* locus.

**Fig. 2:**
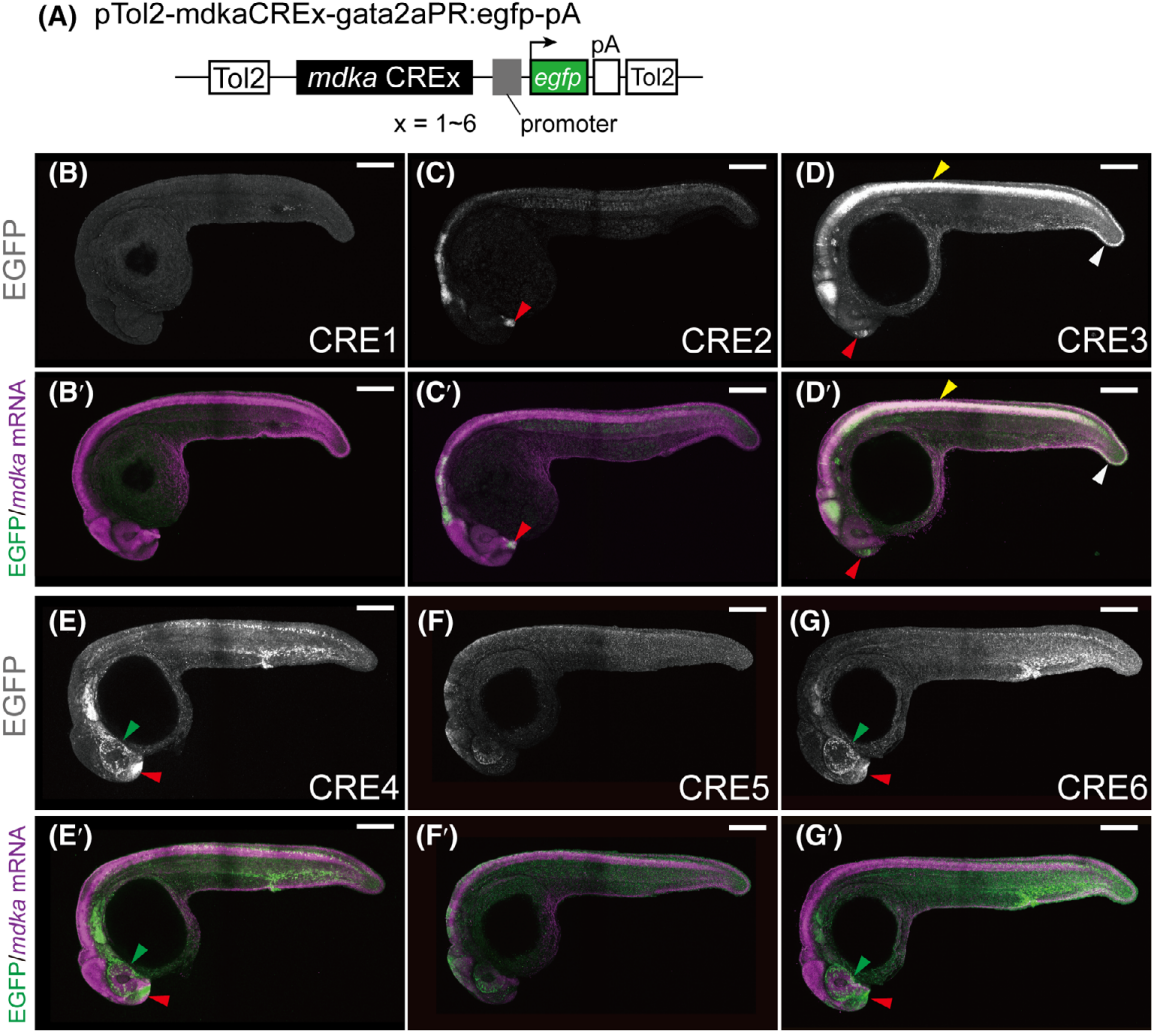
Stable EGFP transgenic line generated using potential *mdka* CREs. (A) Schematic of the plasmid pTol2‐mdkaCREx‐gata2aPR:egfp‐pA, where CREx represents one of the six putative CREs (CRE1 to CRE6). (B–G) Stable EGFP reporter expression driven by *mdka* CRE1 (B), CRE2 (C), CRE3 (D), CRE4 (E), CRE5 (F), CRE6 (G) in embryos at 24 h post‐fertilization (hpf). (B′–G′) Merged images showing *mdka* mRNA (magenta) and EGFP (green) expression in the corresponding CRE transgenic lines. *Tg(CRE3‐gata2aPR:EGFP)* embryos exhibited strong co‐localization of *mdka* mRNA and EGFP signals in the spinal cord (yellow arrowheads, D, D′) and fin fold region (white arrowheads, D, D′). *Tg(CRE4‐gata2aPR:EGFP)* and *Tg(CRE6‐gata2aPR:EGFP)* embryos showed strong activity in the retina (green arrowheads) and brain regions (red arrowheads, E, E′, G, G′), respectively. *Tg(CRE1‐gata2aPR:EGFP)* showed no detectable EGFP expression (B, B′). In panels C, C′ and D, D′, red arrowheads indicate EGFP expression in the brain regions. Representative embryos are shown. Each expression pattern was consistently observed across all established stable transgenic lines (listed in Table S2), each derived from over 150 F₁ adults producing embryos with identical patterns. Scale bar: 200 μm.

To investigate the transcriptional regulation of *mdka*, we conducted a systematic search for CREs controlling *mdka* expression in zebrafish embryos at 24 h post‐fertilization (hpf) and in the ventricular zone of the adult telencephalon under both physiological and injury‐induced conditions. Using data from the DANIO‐CODE repository [14, 37], we identified six potential CREs located upstream, downstream, or in intergenic regions of the *mdka* coding sequence, as shown in Fig. 1. These regions were selected based on chromatin accessibility, as assessed by ATAC‐seq (assay for transposase‐accessible chromatin with sequencing) across zebrafish developmental stages, from dome to long‐pec. We also considered sequence conservation with the carp genome [38], indicated by blue boxes. Based on epigenetic features provided in the DANIO‐CODE resource, including DNA methylation and histone modifications, the potential CREs were classified as promoters or enhancers and categorized by their activation state (repressed, quiescent, or activated) and strength (weak or strong). To validate their regulatory activity *in vivo*, each CRE was cloned upstream of a 1.0‐kb *gata2* minimal promoter, corresponding to the most truncated basal promoter (P5) described by Meng *et al*. [39]. This promoter has been subsequently validated for enhancer assays by Navratilova *et al*. and Zhang *et al*. [25, 40] and was used here to drive EGFP expression (Fig. 2A). These constructs were introduced into the zebrafish germline to generate stable transgenic lines, *Tg(CREx‐gata2aPR:EGFP)*, where CREx represents one of the identified CREs (Fig. 2A). For each construct, we generated at least three independent transgenic lines. All lines for a given construct displayed highly similar EGFP expression patterns, indicating that the observed expression is attributable to the regulatory element rather than positional effects. Notably, none of the CRE1 lines showed detectable expression, whereas five of the six other CREs (CRE2–6) drove EGFP expression in 24 hpf zebrafish embryos, with patterns partially overlapping endogenous *mdka* expression (Fig. 2B–G,B′–G′; Fig. S1A,B). Among them, *Tg(CRE3‐gata2aPR:EGFP)* most closely recapitulated *mdka* expression (Fig. 2D,D′ and Table 1). In this line, EGFP was detected along the spinal cord, tail/fin fold, and brain (Fig. 2D,D′; Table 1; Fig. S1A shows a representative 24 hpf zebrafish embryo with specific anatomical regions). However, none of the individual CREs fully replicated the endogenous *mdka* expression pattern (Fig. 2; Table 1). No EGFP expression was detected in the stable lines generated with CRE1 (Fig. 2B,B′). Several explanations could account for this, including the possibility that CRE1 functions as a silencer. This hypothesis will be briefly discussed in the relevant section. Additionally, each CRE was tested in combination with three different promoters: the *ctgf* promoter [41], the zebrafish synthetic promoter (zsp) [42], and the *mdka* intrinsic promoter (mdkaPR, this study). EGFP expression in these transient transgenics closely resembled that observed using the *gata2a* promoter (Fig. S1C–F), supporting the conclusion that EGFP expression is primarily driven by the CREs rather than by the specific promoter used. Given the consistent expression patterns observed with the *gata2a* promoter, all subsequent characterization of the CREs was carried out using the *gata2aPR:EGFP* construct.

**Table 1:** Summary of EGFP expression patterns driven by individual CREs in 24 h post‐fertilization (hpf) embryos. −, no detectable expression; +, weak expression; ++, strong expression. Expression levels were scored based on both relative signal intensity and spatial coverage, with emphasis on faithfully representing regional differences observed in Fig. 2. Ectopic expression refers to signal detected in tissues that do not exhibit endogenous *mdka* expression (notably CRE4 and CRE6). Additional observations highlighting modularity: (1) CRE3 exhibits strong expression in the midbrain and hindbrain, spinal cord and olfactory tissue, but weaker expression at the hindbrain/spinal cord transition, (2) CRE2 complements this gap with strong expression in the hindbrain and anterior spinal cord, in addition to olfactory tissue, (3) CRE4, CRE5 and CRE6 display robust retinal expression, markedly stronger than that of the other CREs, while retinal activity of CRE3 is comparatively modest, (4) CRE4 and CRE6 display ectopic expression in regions lacking endogenous *mdka* activity, an important property distinguishing these enhancers.

Tissue/region	Endogenous	CRE1	CRE2	CRE3	CRE4	CRE5	CRE6
Forebrain (incl. telencephalon)	++	−	+	+	++	+	++
Midbrain	++	−	+	++	−	+	−
Hindbrain	++	−	++	++	−	+	−
Retina	++	−	+	++	++	+	++
Spinal cord (anterior)	++	−	+	++	+	+	+
Spinal cord (posterior)	++	−	+	++	+	+	++
Notochord	+	−	+	−	−	−	−
Fin fold/Tail	++	−	−	++	+	−	+
Somite/Muscle	++	−	+	−	++	−	++
Ectopic expression	−	−	−	−	++	−	++

### Characterization of CREs driving *mdka* expression in adult NSCs

**Fig. 3:**
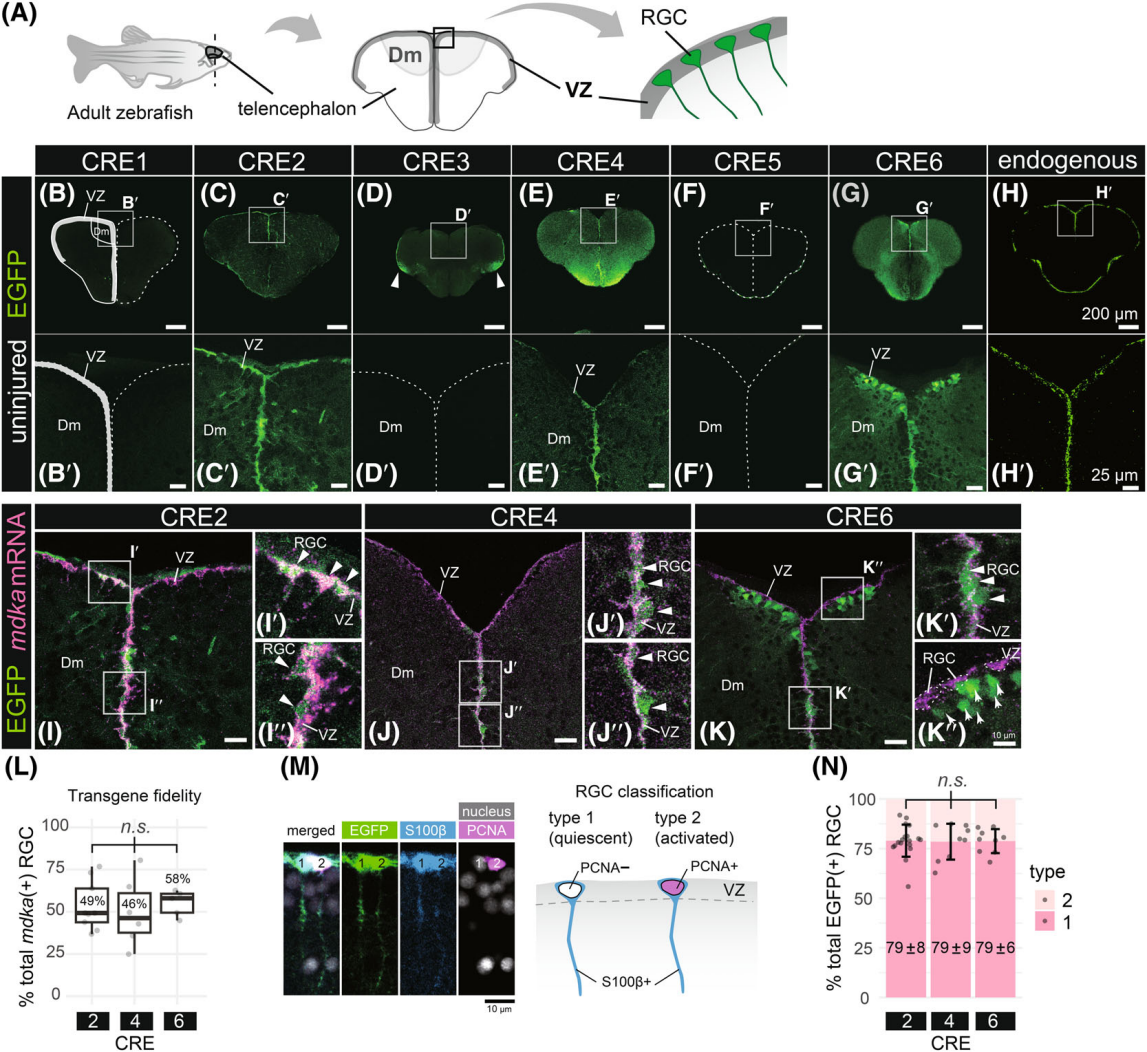
The *mdka* transgenic lines, CRE2, CRE4, and CRE6, drive radial glial EGFP expression in the adult telencephalon during constitutive neurogenesis. (A) Schematic illustrating the orientation of transverse sections through the telencephalon, with reference to the location of radial glial cells (RGCs). Dm: dorsomedial pallium; RGCs: radial glial cells; VZ: ventricular zone. (B–G′) EGFP reporter expression in the adult telencephalon across various *mdka* CRE transgenic lines: CRE1 (B, B′, *n* = 3 brains), CRE2 (C, C′, *n* = 4), CRE3 (D, D′, *n* = 6), CRE4 (E, E′, *n* = 3), CRE5 (F, F′, *n* = 4), and CRE6 (G, G′, *n* = 3). The location of VZ is indicated on the left hemisphere of CRE1 telencephalon, where EGFP expression is absent (B, B′). Rectangles in B–G indicate magnified regions shown in B′–G′, respectively. Dashed lines outline the telencephalon contour (B–G), white arrowheads indicate the posterior dorsal pallium (Dp). (H, H′) Endogenous *mdka* expression in the wild‐type adult telencephalon, shown for reference. Representative images are shown; this expression pattern was consistently observed in at least five independent experiments by the authors and in an additional five experiments by colleagues. I–K and I″–K″ show magnified transverse views of the Dm region, displaying EGFP (green) and *mdka* mRNA expression (magenta) in three transgenic lines with radial glial EGFP expression: *Tg(CRE2‐gata2aPR:EGFP*) (I–I″), *Tg(CRE4‐gata2aPR:EGFP)* (J–J″), and *Tg(CRE6‐gata2aPR:EGFP)* (K–K″). White arrowheads highlight RGCs of *mdka* mRNA and EGFP co‐localization, reflecting faithful activity of CRE2, CRE4 and CRE6. Arrows in the panel K″ indicate the ectopic EGFP expression of CRE6 located outside of VZ. (L) The fidelity of CRE2, CRE4 and CRE6 activity was evaluated by quantifying the percentage of EGFP‐positive cells among endogenous *mdka*‐positive RGCs with the confocal images as shown the panels I–K. Results are shown as boxplots with the median values. No significant difference was observed between different CREs (*n.s*.; *ANOVA* test, *P* = 0.84). (M), S100β + EGFP cells are classified either as type 1 (quiescent, PCNA‐negative) or type 2 (activated, PCNA‐positive) based on PCNA expression. (N) Quantification of type 1 and type 2 RGC population in CRE2, CRE4, and CRE6. The mean ± standard deviation (error bar) of type 1 RGCs is expressed as a percentage of the total RGC population. No significant difference was observed between different CREs (*n.s*.; *Kruskal–Wallis* test, *P* = 0.97). In this panel, EGFP is predominantly expressed in type 1 RGCs (~ 79%) and ~ 21% of type 2 RGCs, closely aligning with Lübke *et al*., who reported endogenous *mdka* expression in 74% of type 1 and 26% of type 2 RGCs. Representative confocal images corresponding to this quantification are provided in Fig. S2A–A‴,B–B‴,A–A‴,C–C‴,D–D‴ showing co‐immunostaining for EGFP, S100β, and PCNA. Each data point represents an individual brain slice. Scale bar: 200 μm (B–H), 25 μm (B′–H′, I–K) and 10 μm (I′–I″, J′–J″, K′–K″ and M). *n* = 3 brains (CRE1; (B), B′), *n* = 6 (CRE2; C, C′, I–I″, (L), N), *n* = 6 (CRE3; D, D′), *n* = 3 (CRE4; E, E′, J–J″, L, N), *n* = 4 (CRE5; F, F′), *n* = 3 (CRE6; G, G′, K, K′, L, N).

In the adult zebrafish telencephalon (schematic representation in Fig. 3A), EGFP expression was detected in four out of six transgenic lines generated (Fig. 3B,B′,C,C′,D,D′,E,E′,F,F′,G,G′) and compared to endogenous *mdka* expression (Fig. 3H,H′). No expression was observed for CRE1 and CRE5 (Fig. 3B,B′,F,F′). CRE3 drove strong ectopic EGFP expression in the posterior dorsal pallium (Dp), as indicated by the white arrowheads, consistent with the subdivisions defined by Schmidt *et al*. [43]. In contrast, no expression was detected in the dorsomedial region (Dm) of the VZ (Fig. 3D,D′). However, CRE2, CRE4, and CRE6 directed EGFP expression in the VZ of the telencephalon, where RGCs reside (Fig. 3C,C′,E,E′,G,G′; Table 2). To determine whether EGFP transgene expression accurately reflects endogenous *mdka* expression, we compared it with endogenous *mdka* expression at single‐cell resolution (Fig. 3H,H′,I–I″,J–J″,K–K″). In CRE2, CRE4, and CRE6 transgenic lines, EGFP‐positive cells were present along the VZ with endogenous *mdka*‐positive RGCs (Fig. 3I–K″). Quantification of confocal stack images showed that roughly half of *mdka*‐positive RGCs co‐expressed EGFP in each CRE transgenic line, with no significant difference among the three CRE lines (Fig. 3L). In the CRE6 transgenic line, EGFP expression was also detected outside of the VZ, where endogenous *mdka* is not expressed, suggesting that CRE6 drives ectopic transgene expression (Fig. 3K″, white arrows). Previously, we reported that endogenous *mdka* expression is enriched in quiescent RGCs (type 1 RGCs; 74% of type 1 cells) compared to activated RGCs (type 2 RGCs; 26% of type 2 cells) [21], we next examined whether CRE2, CRE4, and CRE6 drive EGFP expression in similar manner. To investigate this, we analyzed the expression of the RGC marker S100β and the proliferation marker PCNA in EGFP‐positive cells of each CRE transgenic line (Fig. 3M). All CRE transgenic lines exhibited robust EGFP expression, primarily in quiescent type 1 RGCs marked by S100β+/PCNA−, closely resembling the endogenous *mdka* expression (Fig. 3N; Figs S1G,G′ and S2A–A‴,B–B‴,C–C‴,D–D‴). Although each CRE captured specific aspects of endogenous *mdka* expression, none of them alone fully recapitulated its complete spatial pattern in the adult telencephalon. A similar discrepancy was observed during embryonic development at 24 hpf (Fig. 2). While *Tg(CRE2‐gata2aPR:EGFP)*, *Tg(CRE4‐gata2aPR:EGFP)*, and *Tg(CRE6‐gata2aPR:EGFP)* each reflect certain aspects of *mdka* expression, their activity was incomplete in both spatial distribution and intensity. Interestingly, CREs that were strongly active in the embryonic central nervous system (Fig. 2C,C′) also exhibited activity in the adult telencephalon (Fig. 3C,E,G). However, the spatial expression patterns of these CREs did not fully recapitulate that of endogenous *mdka* mRNA at either developmental stage, indicating that additional regulatory sequences likely contribute to precise *mdka* expression. These findings suggest that the combinations of active CREs required for mdka expression may differ between embryonic and adult tissues, reflecting context‐dependent regulation rather than stage‐specific exclusivity.

**Table 2:** Summary of EGFP expression patterns driven by individual CREs in the adult telencephalon. −, no detectable expression; +, weak expression; ++, strong expression. Dl, lateral pallium; Dm, dorsomedial pallium; Dp, dorsal pallium; Vd, dorsal nucleus of ventral telencephalon; Vv, ventral nucleus of ventral telencephalon; VZ, ventricular zone.

	Endogenous	CRE1	CRE2	CRE3	CRE4	CRE5	CRE6
Dm	++	−	++	−	+	−	+
Dl	++	−	+	+	+	−	+
Dp	+	−	−	++	−	−	−
Vd	+	−	+	−	−	−	−
Vv	+	−	+	−	++	−	−

### Injury‐responsive activity of *mdka* regulatory elements in the adult telencephalon

**Fig. 4:**
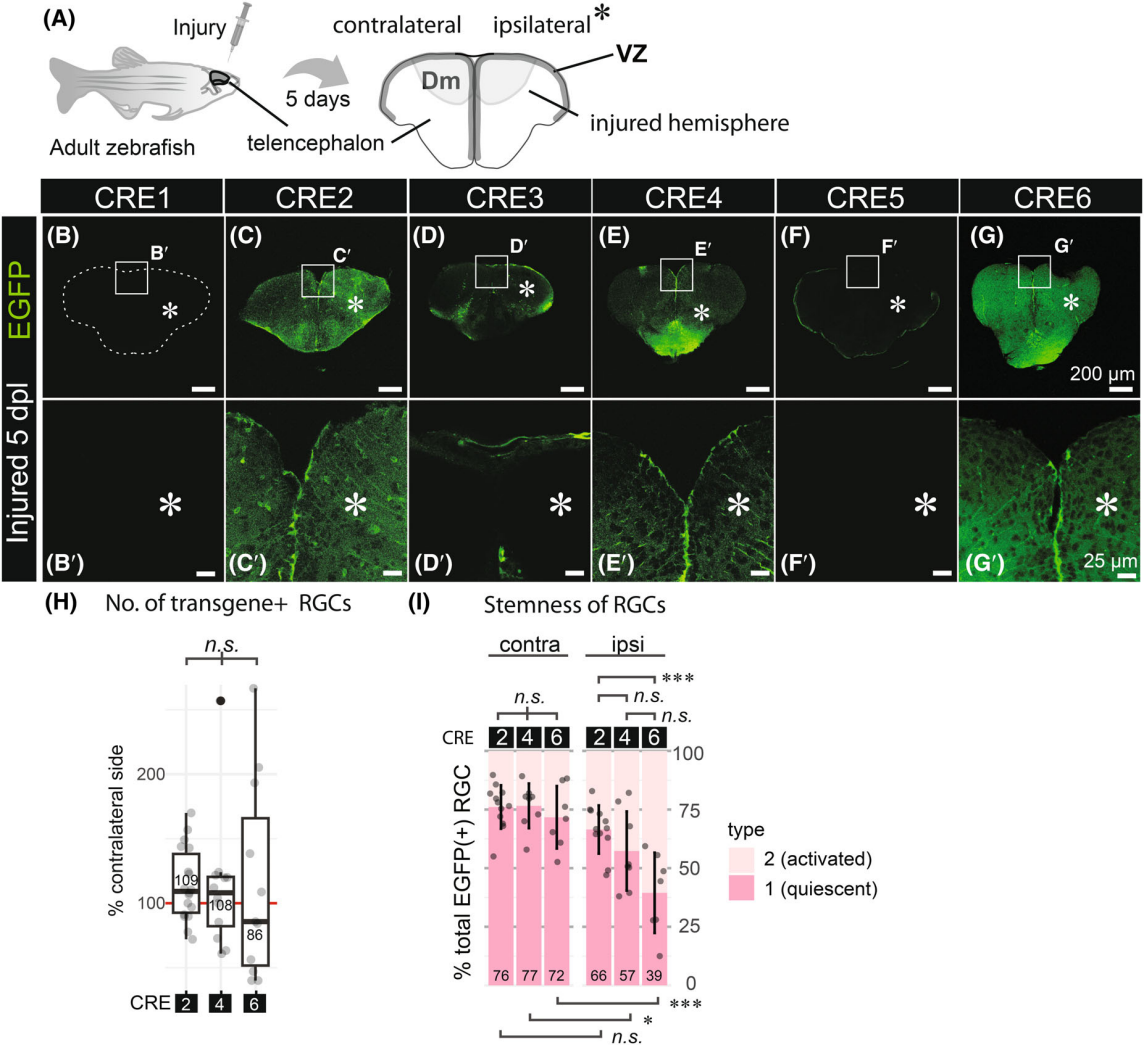
During regenerative neurogenesis, *mdka* CRE2, CRE4 and CRE6, maintain radial glial EGFP expression, but show variability in the injured brain hemisphere. (A) Schematic illustrating the experimental design and orientation of telencephalic brain sections. Adult zebrafish were subjected to stab injury in the right hemisphere of the telencephalon, and brains were collected and fixed at 5 days post‐lesion (dpl). (B–G′) EGFP expression from stable transgenic lines for CRE1 (B, B′), CRE2 (C, C′), CRE3 (D, D′), CRE4 (E, E′), CRE5 (F, F′), and CRE6 (G, G′) is shown for both contralateral and ipsilateral sides (asterisks indicate ipsilateral/injured side) of the telencephalon. Rectangles in B‐G indicate magnified regions shown in B′–G′, respectively. Dashed lines mark the telencephalon contour (B). (H) Quantification of EGFP‐positive and S100β‐positive RGCs. The number of EGFP‐positive RGCs in the injured (ipsilateral) hemispheres of CRE2, CRE4, and CRE6 transgenic lines is shown as a percentage relative to the corresponding contralateral side in boxplots. No significant differences were observed among CREs (*n.s*.; *ANOVA*, *P* > 0.05). (I) Stemness of EGFP‐positive RGCs in CRE2, CRE4, and CRE6 was assessed. Relative proportions of type 1 (quiescent, S100β^+^/PCNA^−^) and type 2 (activated, S100β^+^/PCNA^+^) RGCs in contralateral and ipsilateral hemispheres are presented as stacked bar plots. While no statistical differences were found among CREs in the contralateral hemisphere, a significant difference was observed between CRE2 and CRE6 in the ipsilateral (injured) hemisphere (*ANOVA* followed by *Tukey*'s test, *P* < 0.001, ***; error bars represent mean ± standard deviation). Representative confocal images used for quantifications in panels H and I are provided in Figs S3, showing co‐immunostaining for EGFP, S100β, and PCNA. Each data point represents an individual brain slice. Scale bars: 200 μm (B‐G); 25 μm (B′‐G′). Sample sizes: *n* = 3 brains (B, B′), *n* = 4 (C, C′), *n* = 5 (D, D′), *n* = 3 (E, E′), *n* = 4 (F, F′), *n* = 3 (G, G′), *n* = 3 for each CRE (H, I).

Since *mdka* expression in the ventricular zone is upregulated during the regenerative response following injury particularly in type 1 RGCs (1.37‐fold increase, *P* = 2.68 × 10^−9^) [21], we subjected all six *mdka* CRE transgenic lines to telencephalic injury and analyzed their expression patterns in these conditions (Fig. 4A,B–G,B′–G′; Figs S1H,H′ and S3A–A″,B–B‴,C–C‴,D–D‴). All injury‐related analyses were performed at 5 days post‐lesion (dpl), the time point at which *mdka* expression peaks in the injured hemisphere according to previous RNA‐seq and RT‐qPCR data [21]. This time point therefore provided an optimal window to evaluate injury‐induced regulatory activity of the CREs. The injury was induced in the right hemisphere using a 30G syringe needle inserted through the skull [27, 44], while the uninjured contralateral hemisphere served as an internal control (Fig. 4A). Following injury, EGFP reporter expression remained absent in the VZ of the Dm region in transgenic lines carrying CRE1, CRE3, and CRE5 (Fig. 4B,B′,D,D′,F,F′), consistent with the lack of expression observed under homeostatic conditions (Fig. 3B,B′,D,D′,F,F′). In contrast, transgenic lines with CRE2 (Fig. 4C,C′), CRE4 (Fig. 4E,E′), and CRE6 (Fig. 4G,G′) showed EGFP expression in the Dm of the VZ. However, for all three CREs, the number of EGFP‐positive RGCs in the injured hemisphere remained largely unchanged compared to the uninjured contralateral side, with no significant differences between the CREs (Fig. 4H). Assessment of RGC stemness, based on the proportion of type 1 RGCs among EGFP‐positive cells, revealed differences between CREs in the injured (ipsilateral) brain hemisphere. Specifically, CRE2 showed a significantly higher proportion of quiescent type 1 RGCs compared to CRE6 (Fig. 4I, *P* < 0.01). In contrast, on the contralateral (uninjured) side, approximately three‐quarters of EGFP‐positive RGCs remained quiescent across all CRE lines, consistent with the proportions observed under homeostatic conditions (Figs 3N and 4I). These results suggest that none of the individual CREs contain a strongly injury‐responsive regulatory element sufficient to upregulate *mdka* expression during regeneration. Similar to the regulation observed under physiological conditions, it is likely that multiple CREs must act in combination to mediate a full regenerative response.

### Cooperative CRE activity enhances faithful transgene expression in embryos, adult RGCs, and in response to injury

**Fig. 5:**
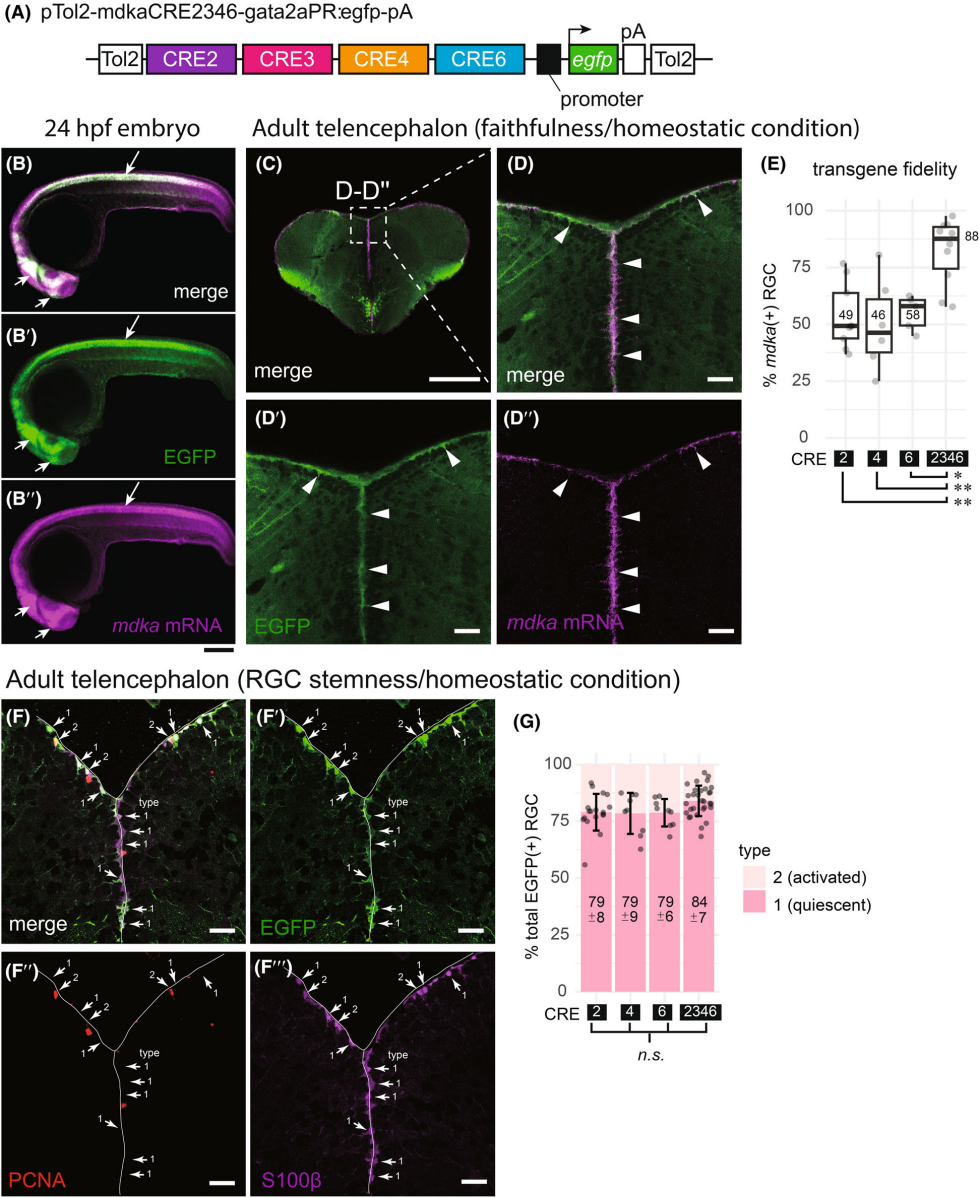
Combination of CREs enhances faithful transgene expression in both embryos and the adult telencephalon under homeostatic conditions. (A) Schematic of the pTol2‐mdkaCRE2346‐gata2aPR:egfp‐pA construct, in which a combination of CRE2, CRE3, CRE4, and CRE6 drives EGFP expression initiated by the *gata2a* promoter region (*gata2aPR*). (B–B″) Comparison of EGFP transgene (B′) and *mdka* mRNA (B″) expression in 24 h post‐fertilization (hpf) embryo. Merged EGFP and *mdka* mRNA signals (B) show co‐localization primarily in the brain and spinal cord (white arrows). (C) Merged EGFP and *mdka* mRNA expression in a transverse section of the adult telencephalon under homeostatic conditions. The magnified Dm region (dashed rectangle) is shown in D–D″. (D–D″) Magnified views of the telencephalic Dm domain showing merged signals (D), EGFP driven by CRE2346 (green, D′), and endogenous *mdka* mRNA (magenta, D″). White arrowheads indicate RGCs in the ventricular zone (VZ). (E) Faithfulness of EGFP transgene expression driven by CRE2, CRE4, CRE6, and the combined CRE2346. EGFP expression in VZ of each CRE transgenic line was compared with endogenous *mdka* mRNA. Data are shown as boxplots representing the percentage of EGFP^+^/*mdka* ^+^ double‐positive cells relative to total *mdka* ^+^ cells. Median values are indicated. Significant differences were observed between CRE2346 and individual CREs (*ANOVA* followed by *Tukey's* test; CRE2346 vs CRE2 [*P* < 0.01], vs CRE4 [*P* < 0.01**], vs CRE6 [*P* < 0.05*]). The data for individual CREs are identical to that shown in Fig. 3L. (F–G) Stemness analysis of RGCs labeled by CRE2346‐driven EGFP. EGFP‐positive cells in the VZ of the telencephalic Dm domain were categorized as type 1 (quiescent, S100β^+^/PCNA^−^) or type 2 (activated, S100β^+^/PCNA^+^) using immunolabeling. (F–F‴) Transverse telencephalon sections showing EGFP (F′), PCNA (F″), S100β (F‴), and merged image (F), highlighting type 1 and 2 RGCs under homeostatic conditions. (G) Stemness of CRE2346‐driven EGFP‐positive RGCs was assessed and compared to those of individual CREs in a stacked bar chart. The data for individual CREs are reused from Fig. 3N to facilitate comparison. Mean ± standard deviation (SD) of type 1 RGCs (as a proportion of EGFP‐positive RGCs) is shown. Each data point represents an individual brain slice. No significant differences were observed among different CREs (*n.s*.; *Kruskal–Wallis* test, *P* = 0.05). Representative embryo images are shown; identical expression patterns were observed in all four independent stable CRE2346 transgenic lines (listed in Table S2). Scale bars: 200 μm (B–B″ and C); 25 μm (D–D″, F–F‴). Sample sizes: *n* = 3 brains (C, D–D″, E); *n* = 5 brains (F–F‴, G).

Analysis of individual stable transgenic lines showed that none of the six CREs alone fully recapitulated the spatial expression pattern of *mdka* throughout embryonic development and adulthood, under either homeostatic or regenerative conditions. To investigate potential cooperative interactions among these CREs, we generated a combined CRE2346 transgenic reporter construct (Fig. 5A). This construct integrates CRE3, which showed strong regulatory activity during embryogenesis, together with CRE2, CRE4, and CRE6, which were effective in the adult telencephalon. Under homeostatic conditions, the CRE2346 construct drove EGFP expression patterns that closely mimicked endogenous *mdka* expression in embryos (Fig. 5B–B″). In the adult telencephalon (Fig. 5C,D–D″), CRE2346 mediated specific EGFP expression in the Dm of the VZ (white arrowheads, Fig. 5D), displaying RGC morphology and co‐expression with *mdka* endogenous expression. Transgene fidelity assessment revealed that CRE2346 was active in the majority (median 88%) of *mdka*‐positive RGCs, demonstrating significantly higher coverage than any individual CRE (Fig. 5E). However, analysis of RGC stemness, assessed by immunolabeling for PCNA and S100β, showed no significant effect of the CRE combination (Fig. 5F–F‴,G).

**Fig. 6:**
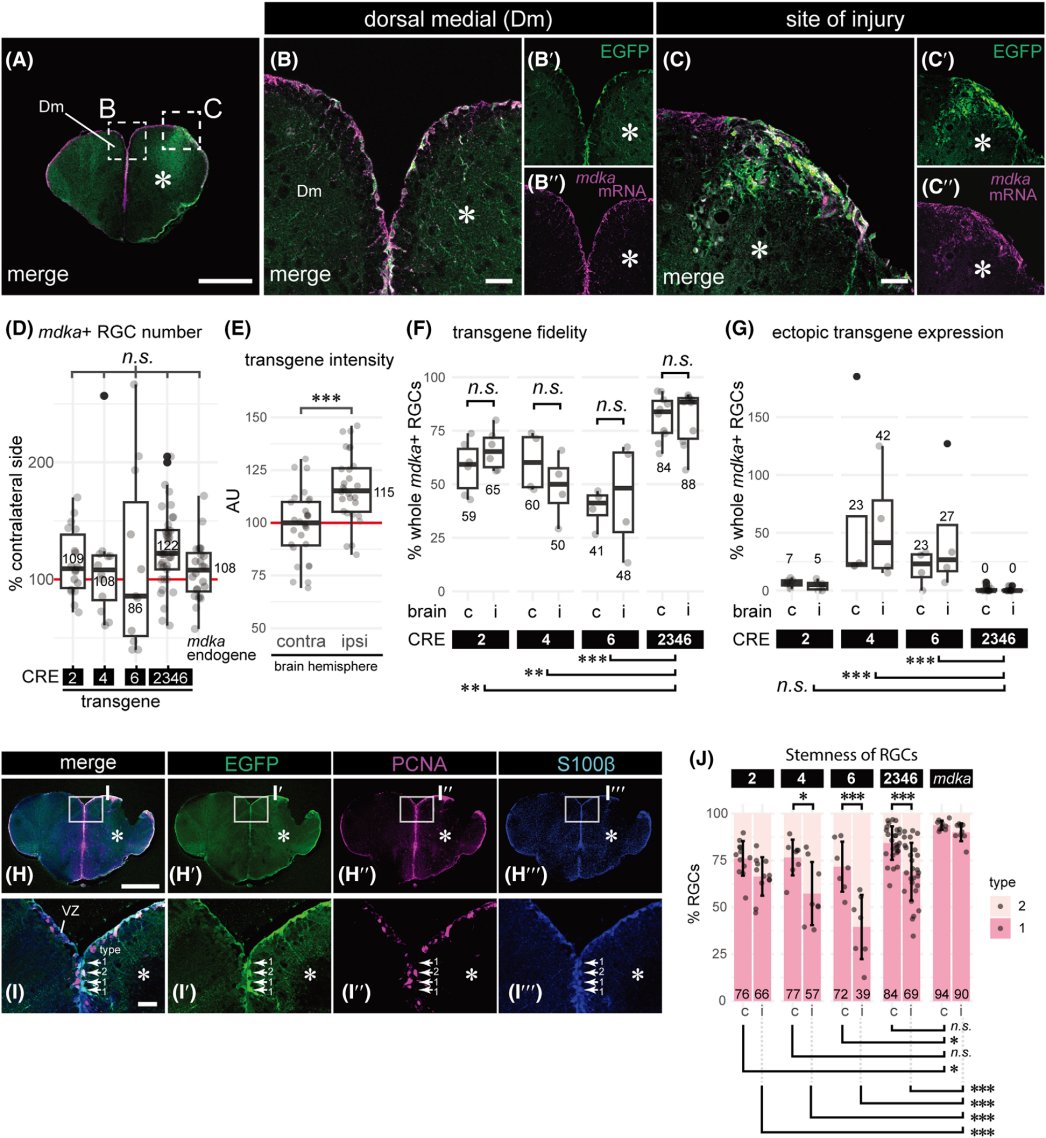
Combination of CREs enhanced faithful transgene expression in the adult telencephalon after brain injury. (A–C″) Merged images of EGFP expression driven by CRE2346 (green) and endogenous *mdka* mRNA (magenta) in a transverse section of the adult telencephalon during regenerative neurogenesis at 5 days post‐lesion (dpl). The hemisphere ipsilateral to the injury is marked with an asterisk. (A) Overview of the telencephalon. Stippled rectangular regions indicate areas magnified in B–B″ (Dm domain) and C–C″ (site of injury). (B–B″) Magnified merged image of CRE2346‐driven EGFP (green, B′) and endogenous *mdka* mRNA (magenta, B″). (C‐C′) Magnified merged view at the site of injury (C), showing upregulated expression of CRE2346‐driven EGFP (green, C′) and endogenous *mdka* mRNA (magenta, C″). (D) The number of S100β‐positive RGCs expressing either CRE transgenes or endogenous *mdka* mRNA in the hemisphere ipsilateral to the injury did not differ between CRE constructs and the endogenous gene (*n.s*.; *Kruskal–Wallis* test, *P* = 0.11). The number of ipsilateral RGCs is normalized to that of contralateral RGCs. Horizontal red line indicates RGC count on the contralateral side. (E) In the CRE2346 transgenic line, EGFP levels in the ipsilateral hemisphere are significantly higher than in the contralateral side (*Welch t*‐test, *P* < 0.001***). Integrated reporter fluorescence in the ipsilateral hemisphere was normalized to the contralateral side, set to 100 arbitrary units (AU; red line). (F–G) Faithfulness of (F) and ectopic (G) EGFP transgene expression driven by either CRE2, CRE4, CRE6 or CRE2346 in contralateral (“c”) and ipsilateral hemispheres (“I”) after brain injury. (F) EGFP expression in VZ of each CRE transgenic line were compared with endogenous *mdka* mRNA expression. Results are presented in boxplots showing the percentage of cells double‐positive for EGFP and endogenous *mdka* mRNA relative to the total number of *Stemness*‐positive cells. Significant differences were observed between CRE2346 and each individual CREs (*Dunn's post hoc* test after *Kruskal–Wallis*: CRE2346 vs CRE2 [*P* < 0.01**], vs CRE4 [*P* < 0.01**] and vs CRE6 [*P* < 0.001***]). No significant difference was observed between contralateral and ipsilateral hemispheres in all cases (*n.s*.; *Dunn's post hoc* test after *Kruskal–Wallis* analysis). (G) Ectopic EGFP expression outside the VZ of each CRE transgenic line are shown relative to the number of endogenous *mdka*‐positive RGCs. Although no significant difference was observed between contralateral and ipsilateral hemispheres, CRE2346 showed significantly lower ectopic transgene expression compared to CRE4 (adjusted *P*‐values in *Dunn* test following *Kruskal–Wallis* test; *P* < 0.001***) and CRE6 (*Dunn* test following *Kruskal–Wallis* test; *P* < 0.001***). H‐J, Evaluation of RGC stemness in the CRE2346 line using PCNA and S100β immunostaining. (H–H‴, I–I‴) Transverse section of the adult telencephalon during regenerative neurogenesis at 5 dpl, showing the merged image (H–I) of CRE2346‐driven EGFP (green, H′–I′), PCNA (magenta, H″–I″) and S100β (blue, H‴–I‴). The white rectangular regions in H–H‴ are magnified in I–I‴, respectively. Representative type 1 (S100β^+^/PCNA^−^) and type 2 (S100β^+^/PCNA^+^) RGCs are indicated (I–I‴). (J) Stemness of CRE2346‐driven EGFP‐positive RGCs compared to individual CRE lines and endogenous *mdka* mRNA (“*mdka*”). Pairwise comparisons were performed using *Dunn*'s test following a significant *Kruskal–Wallis* test (*n.s*. for *P* > = 0.05, * for *P* < 0.05 and *** for *P* < 0.001, error bars represent mean ± standard deviation). The results for hemispheres contralateral (“C”) or ipsilateral (“I”) to the injury are plotted adjacent to each other. The data for CRE2, CRE4, and CRE6 are identical to Fig. 4I. Each data point represents an individual brain slice. Black solid dots indicate outliers. Scale bars: 200 μm (A, H–H‴); 25 μm (B–B″, C–C″, I–I‴). Sample sizes: *n* = 4 brains (A, B–B″, C–C″); *n* = 6 brains (CRE2346; (D), H–H‴, I–I‴, J); *n* = 3 brains (E–G); *n* = 3 brains (endogenous expression of *mdka*; D, J).

We next investigated how the combination of *mdka* cis‐regulatory elements (CREs) influences transgene expression during regenerative neurogenesis following brain injury (Fig. 6). After stab injury, EGFP expression driven by the CRE2346 construct was observed in the ventricular zone (VZ) of both the injured (ipsilateral) and uninjured (contralateral) hemispheres, closely mirroring the endogenous *mdka* expression pattern (Fig. 6A,B–B″). Notably, injury‐induced *mdka* upregulation at the lesion site, which occurs outside the VZ, was also faithfully reproduced by the CRE2346 line (Fig. 6C–C″). Quantification revealed that the number of radial glial cells (RGCs) expressing EGFP in the injured hemisphere was comparable to that in the contralateral side across all CRE lines, further reflecting endogenous *mdka* expression (Fig. 6D). Moreover, EGFP intensity was significantly increased in the injured hemisphere in the CRE2346 line (*P* < 0.001), aligning with our prior observations of injury‐induced *mdka* upregulation (Fig. 6E) [21]. To evaluate the impact of combining CREs, we compared transgene fidelity and ectopic expression between CRE2346 and single CRE lines (Fig. 6F,G). The combination significantly improved transgene fidelity, with consistent expression levels between ipsilateral and contralateral hemispheres (Fig. 6F). Additionally, CRE2346 effectively suppressed the ectopic, off‐target expression seen with CRE4 and CRE6 alone, which had previously shown high levels of nonspecific activity (Fig. 6G). We then assessed whether the CRE combination preserved the identity and stemness of transgene‐expressing RGCs. Co‐immunolabeling with S100β and PCNA allowed us to distinguish quiescent (type 1) from activated (type 2) RGCs (Fig. 6H–H‴,I–I‴). In the uninjured (contralateral) hemisphere, CRE2346 increased the proportion of EGFP+ quiescent RGCs to 84%, closely approaching the 94% observed for endogenous *mdka* expression (Fig. 6J). However, in the injured hemisphere, this fidelity was reduced. All constructs, including CRE2346, showed a marked decrease in the proportion of quiescent RGCs (39–69%), significantly lower than the 90% observed with endogenous *mdka*. This suggests that although the CRE2346 line closely replicates endogenous *mdka* activity under normal conditions and partially during regeneration, it likely lacks additional regulatory elements necessary for fully recapitulating *mdka* injury response, particularly for maintaining the quiescent RGC state.

**Fig. 7:**
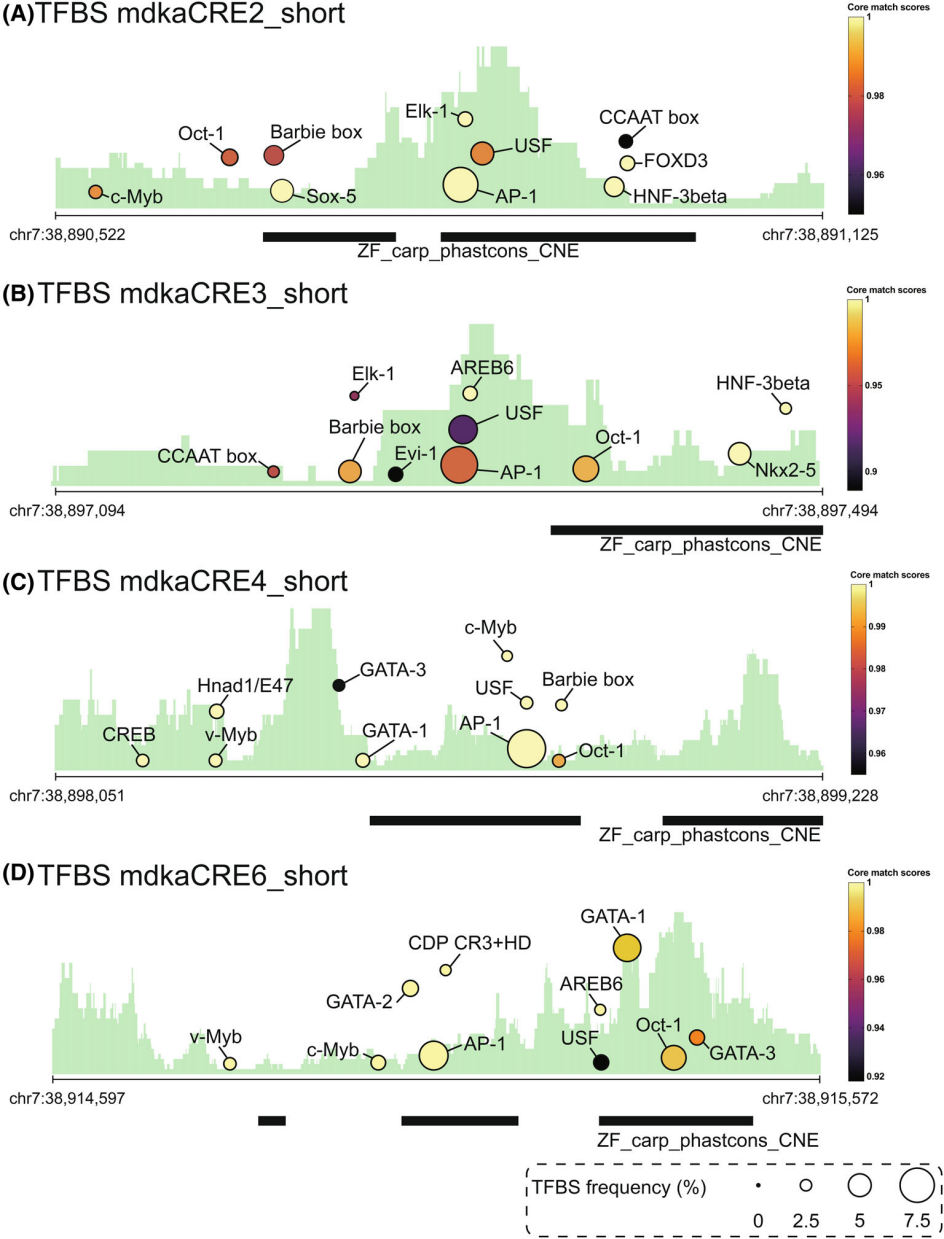
Potential transcription factor binding motifs identified in four *mdka* CREs. Top 10 transcription factors binding sites (TFBS) were identified in the short versions of CRE2 (A), CRE3 (B), CRE4 (C), and CRE6 (D). Prominent motifs include AP‐1, Oct‐1, and USF. The CREs were predicted using ATAC‐seq, ChIP‐seq data (green background profiles; H3K27ac, 90 days to 2 years brain data from Shkumatava Lab, accessible via NCBI GEO accession GSE75734), and evolutionary conservation data (ZF_carp_phastcons_CNE). Circle color and size indicate the strength of TF matches and motif prevalence. Genomic coordinates for each short CRE are provided according to the GRCz10 assembly (e.g., chr7:38890522–38891125 for CRE2).

In summary, our findings demonstrate that the CRE2346 combination effectively drives *mdka*‐like EGFP expression in RGCs with spatial specificity and responsiveness to injury (Table 3). This highlights the necessity of multiple regulatory elements for precise spatial and temporal control of *mdka* expression under both physiological and regenerative conditions. Interestingly, all four CREs contain multiple transcription factor binding sites, including robust AP‐1 binding sites within their evolutionarily conserved regions (Fig. 7A–D). AP‐1 is a well‐known regulator of gene expression during regeneration across various tissues and species, including the zebrafish heart, fin, and tail [35, 45, 46, 47, 48, 49], supporting the potential role of these CREs in the regenerative response to injury.

**Table 3:** Summary of EGFP expression patterns observed in distinct *mdka* CRE transgenic lines. Dl, lateral pallium; Dp, dorsal pallium; NSCs, neural stem cells.

CRE(s)	Activity summary
CRE2	Adult regulatory sequence; active in physiological conditions; best matching *mdka* expression
CRE3	Predominantly active during embryonic development; overlaps with mdka expression; shows ectopic expression in the adult telencephalon in Dp and Dl
CRE4	Adult regulatory sequence; active in NSCs; does not show a significant response to injury
CRE6	Adult regulatory sequence; active in NSCs with ectopic activity in neurons; no significant response to injury
CRE2346	Recapitulates *mdka* expression; EGFP reporter overlaps with *mdka* mRNA in both embryo and adult; inducible upon injury

## Discussion

Our study reveals that *mdka* expression in the zebrafish central nervous system is regulated by a complex and highly context‐dependent network of CREs. These elements act in a modular, combinatorial, and sometimes redundant fashion, reflecting a multifaceted regulatory architecture that governs *mdka* transcription across different developmental stages and physiological states. Consistent with previous reports, *mdka* displays dynamic, tissue‐specific expression patterns. For example, in the adult zebrafish telencephalon, *mdka* is expressed in RGCs under homeostatic conditions [21], while in the retina, it is absent in resting stem cells but becomes induced in proliferating Müller glia following injury [29, 50]. Interestingly, in the injured telencephalon, *mdka* expression remains restricted to quiescent RGCs, underscoring its tight, tissue‐specific transcriptional control [21].

### Modular and context‐specific regulation of *mdka*

Our transgenic analyses demonstrate that *mdka* expression is orchestrated by multiple CREs, each contributing distinct spatiotemporal aspects to its regulatory program. Some elements, such as CRE5 and CRE3, are active primarily during embryogenesis. For example, CRE3 drives strong expression in the embryonic brain and spinal cord but fails to recapitulate specific adult brain patterns, instead causing ectopic and nonspecific activation (Table 3). In contrast, elements like CRE2 are active mainly in the adult telencephalon, particularly in RGCs located in the Dm of the VZ. This modularity is not limited to the brain. In the heart, previous studies showed that a transgene containing *mdka* intronic sequences (*mdka*_e1, overlapping our CRE3, Table S1) was active in the developing epicardium but inactive after adult injury [35]. Meanwhile, a downstream element (*mdka*_e2, overlapping CRE6, Table S1) was inactive during development but became induced in a subset of adult epicardial cells following injury [35]. These examples reinforce the idea that distinct CREs mediate *mdka* expression in different developmental or physiological contexts.

### Combinatorial input cooperative and redundant CRE activity

While individual CREs contribute to specific features of *mdka* expression, none alone were sufficient to fully recapitulate endogenous expression, particularly in response to injury. For instance, CRE2, CRE4, and CRE6 each drove weak expression in adult telencephalic RGCs, but none showed injury responsiveness on their own. However, when combined in a multi‐CRE construct, these same elements collectively drove expression that closely mimicked endogenous *mdka*, including robust injury‐induced activation. This supports a model in which full *mdka* expression depends on the cooperative action of multiple CREs. Such combinatorial regulation may reflect the integration of distinct signaling inputs across separate regulatory elements. One possibility is that different signaling pathways act through specific CREs, with their additive or synergistic activity required to achieve the proper spatiotemporal expression profile. In this context, the effects of different CREs may be additive at the tissue level, such that multiple enhancers with overlapping activity collectively generate higher overall expression than any single element. Mechanistically, at the single‐cell level, promoter–enhancer interactions are typically limited to one allele per cell, with biallelic expression allowing activity from up to two alleles of the endogenous *mdka* gene. For transgenes, copy number and zygosity may vary, which can also influence the expression levels observed across the tissue. Thus, the apparent additive effect likely reflects the summation of expression across multiple cells rather than multiple enhancers simultaneously acting on a single allele, a distinction that we now clarify. This combinatorial strategy may also contribute to enhanced specificity by limiting enhancer activity to overlapping spatial domains, thereby reducing ectopic expression. The presence of conserved AP‐1 binding motifs within CRE2, CRE3, CRE4, and CRE6 (Fig. 7A–D), coupled with the established role of the AP‐1 complex in regeneration [34, 45, 48], supports both scenarios. Importantly, our observations also reveal redundancy among CREs. Although each element has specific functions, some show overlapping activity. For instance, despite weak individual responses to injury, CRE2, CRE4, and CRE6 all target the same VZ region. Furthermore, CRISPR/Cas9‐mediated deletion of a regulatory element in the regenerating fin did not abolish *mdka* expression, and regeneration proceeded without detectable patterning defects [34]. This suggests that redundant or compensatory enhancer activity may preserve gene expression when individual elements are disrupted. Such redundancy adds robustness to the regulatory system, ensuring consistent *mdka* activation even in fluctuating or damaged environments. Together, our results highlight a regulatory logic in which enhancer modularity, cooperation, and redundancy work in concert to control *mdka* expression with high precision and resilience.

### Broader implications for gene regulation and regenerative biology

The modular and distributed regulatory architecture of *mdka* is consistent with classical models of enhancer function. The *even‐skipped (eve)* gene in *Drosophila*, for example, is expressed in a seven‐stripe pattern during embryogenesis, with multiple enhancers contributing to this pattern; some enhancers control expression in more than one stripe by integrating local signals [51, 52]. Similarly, in mammals, the neural crest gene *sox10* is regulated by multiple enhancers with overlapping and redundant functions, enabling robust expression across tissues and stages [53, 54]. These cases, like *mdka*, illustrate how enhancer modularity enables nuanced, context‐dependent expression and buffering against perturbation. Our *mdka* reporter lines provide a useful system for dissecting transcriptional programs that govern adult neurogenesis and injury‐induced gene activation. These tools could be employed to identify upstream regulators of regenerative gene expression and screen for factors that modulate regenerative capacity *in vivo*.

### Regulatory complexity vs. simplicity: a comparative perspective

Intriguingly, the complexity of *mdka* regulation contrasts with the simpler enhancer architecture observed for other genes expressed in the same cellular context. For example, *id1*, which is co‐expressed with *mdka* in quiescent RGCs under both physiological and regenerative conditions, appears to be controlled by a single enhancer responsive primarily to BMP signaling [24, 25]. Despite their overlapping expression domains, *id1* and *mdka* rely on very different regulatory strategies. This contrast raises important questions: Why do some genes require complex enhancer networks while others are regulated through simpler mechanisms? Comparative studies between genes like *mdka* and *id1* could illuminate how enhancer complexity evolves and functions in the context of regeneration and plasticity.

### Limitations and future directions in defining the *mdka* regulatory landscape

Our primary goal was to identify and functionally validate CREs that positively regulate *mdka* expression. Using *in vivo* transgenesis in zebrafish embryos and adults, we discovered several elements that drive EGFP expression in patterns consistent with endogenous *mdka* activity. These findings provide a foundation for understanding how *mdka* is regulated during development and regeneration. However, a key limitation of our approach is that it is optimized to detect enhancer activity and does not capture negative regulatory elements such as silencers. Traditional reporter assays are unable to distinguish between a lack of enhancer function and active repression. For instance, CRE1 did not drive detectable EGFP expression on its own, but whether this indicates silencer activity or simply absence of regulatory function remains unresolved. To address this, future studies should explore combinatorial testing, such as integrating CRE1 with active enhancers like CRE2346, to determine if it can restrict or suppress ectopic expression. Such designs could uncover context‐dependent repression that would remain hidden when elements are tested in isolation. Our data also underscore the modular nature of the *mdka* regulatory architecture, which involves synergy, additivity, and redundancy among CREs. Additive effects, where multiple elements with overlapping activity are needed to achieve full expression, should be distinguished from redundancy, where one CRE can compensate for the absence of another. Both features likely contribute to the robustness and precision of *mdka* expression in both physiological and regenerative contexts. A fuller understanding of this regulatory logic will require experimental strategies that can reveal both positive and negative regulatory interactions.

Another limitation concerns the use of fluorescent reporter systems. The relative stability of EGFP protein may lead to a prolonged signal even after *mdka* mRNA expression has declined, potentially contributing to discrepancies in the timing of spatial localization. To address this, we performed *in situ* hybridization for *egfp* mRNA, which confirmed both expected expression in the dorsomedial region and ectopic expression in the parenchyma (data not shown). These findings further support the idea that certain silencer elements may be absent from our current constructs. Given its lack of activity in isolation, CRE1 remains a strong candidate for a negative regulatory element, and its function in combination with other enhancers will be a priority for future studies. In addition, mRNA‐level analysis across multiple transgenic lines will be essential for identifying those that most accurately recapitulate endogenous *mdka* expression in radial glial cells. Collectively, these insights highlight the importance of both enhancer and silencer elements in shaping the spatial and temporal dynamics of *mdka* regulation under homeostatic and regenerative conditions.

## Materials and methods

### Zebrafish husbandry

All zebrafish lines were maintained at the European Zebrafish Resource Center (EZRC). Experiments were performed on 6‐ to 12‐month‐old wild‐type fish or transgenic reporter lines (Table S2). Animal husbandry was carried out in accordance with permit guidelines under §11 TSchG (Regierungspräsidium Karlsruhe, Aktenzeichen 35‐9185.64/BH KIT). Fish were kept according to the European Society for Fish Models in Biology and Medicine (EuFishBioMed) guidelines at the Karlsruhe Institute of Technology (KIT) [55], and animal experimentation adhered to German animal protection standards, approved by the Government of Baden‐Württemberg (Regierungspräsidium Karlsruhe, Reference Numbers: 35‐9185.81/G‐214/21 and 35‐9185.81/G‐215/21). Efforts were made to minimize animal distress and reduce their number.

### Plasmids construction

Plasmids for enhancer/promoter reporter assays were generated using Gateway cloning and TOPO/TA cloning strategies. The primers used for PCR amplification of putative CREs and promoter are listed in Table S3, while the coordinates and sizes of the CREs and the promoter are provided in Table S1. Most plasmids (Table S4) were constructed using commonly available vectors, with detailed protocols for Gateway cloning available as described in Kwan *et al*. [56]. The pTol2‐mdkaCRE2346‐gata2aPR:egfp‐pA plasmid was created by combining four *mdka* putative CREs (CRE2, CRE3, CRE4, and CRE6) into the pT2KHGPzGATA2C1 destination vector and was synthesized by GenScript Biotech Corporation (Rijswijk, Netherlands).

### Microinjection to generate transient and stable transgenesis

One‐cell stage embryos were injected with 1 μL of 20 ng·μL^−1^ Tol2 transposase mRNA, 1 μL of 10% phenol red, 50 ng·μL^−1^ plasmid DNA, and nuclease‐free water (final volume 10 μL) directly into the yolk through the chorion. After injection, embryos were incubated at 28 °C in E3 medium with methylene blue. At 24 hpf, embryos were sorted based on EGFP fluorescence. Positive F0 founders were identified by EGFP expression, raised to sexual maturity, and outcrossed with wild‐type zebrafish. Stable EGFP expression in ~ 20% of F1 progeny confirmed successful stable transgenesis.

### Fluorescent *in situ* hybridization (FISH) of whole‐mount embryos

Embryos were collected at the desired developmental stage, and chorions were removed manually or with pronase (1 : 100 w/v, 28.5 °C). The *mdka* probe (Molecular Instruments Inc., Los Angeles, CA, USA) was used, following the HCR™ RNA‐FISH protocol with minor modifications, available on the Molecular Instruments website.

### Stab injury of adult telencephalon and preparation of sections

The stab injury experiment and telencephalon sections were performed following the protocol described by Schmidt *et al*. [44].

### FISH on adult telencephalon sections and embryos

For experiments using probes from Molecular Instruments Inc., the protocol “HCR™ RNA‐FISH protocol for fresh frozen or fixed frozen tissue sections” was followed, with minor modifications. Specifically, for 24 hpf embryos, proteinase K treatment was conducted at a concentration of 10 μg·mL^−1^ (diluted from a 1000× stock to 1× working concentration) for 10 min to improve probe penetration. The *mdka* probe used for fluorescent *in situ* hybridization was purchased from Molecular Instruments Inc. with a dilution of 1 : 500.

### Immunohistochemistry (IHC) on adult telencephalon sections

Vibratome sections were blocked with 1 mL blocking buffer for 1 h at RT. After removing the buffer, sections were incubated overnight at 4 °C with primary antibodies in 300 μL blocking buffer. The next day, sections were washed 3 × 10 min with PTW (1× PBS containing 0.1% Tween‐20), then incubated with secondary antibodies (diluted in PTW) for 2 h at RT in the dark. DAPI staining (1 : 2000 in PTW) was performed for 20 min if needed, followed by 3 × 5 min washes in PTW. Sections were mounted and imaged. All antibodies used are listed in Table S5.

### Mounting for imaging

For embryos, dechorionated embryos were embedded in a 1.6% low melting‐point agarose (Sigma‐Aldrich Chemie GmbH, Taufkirchen, Germany) and 0.016% Tricaine (Sigma) mixture in E3 medium, oriented within 1 min using a needle or fine hair, left at room temperature for 3 min, cooled at 4 °C for 3 min, and supplemented with water drops to prevent evaporation. While for adult telencephalon sections, fluorescently labeled brain sections were mounted on glass slides with 0.17 mm coverslips using Aqua Polymount (PolySciences Inc., USA) and stored at 4 °C in the dark before and after imaging [44].

### Confocal imaging

Fluorescently labeled embryos and telencephalon sections were imaged using Leica TCS SP5 DM5000 and SP8/DLS confocal fluorescence microscopes (Leica Microsystems, Wetzlar, Germany) equipped with 405 nm, 488 nm, 561 nm, and 633 nm lasers. Excitation (Ex) wavelengths and emission (Em) ranges were as follows: Ex 488 nm for AlexaFluor 488 (EGFP, Em 492–550 nm), Ex 561 nm for AlexaFluor 561 (PCNA, Em 565–605 nm), Ex 633 nm for AlexaFluor 633 (S100β, Em 650–740 nm), Ex 633 nm for AlexaFluor 633‐labeled *mdka* RNA probe (Em 655–724 nm), and Ex 405 nm for DAPI (Em 409–480 nm). Imaging was performed with sequential scanning at 16‐bit color depth with a 1.0 AU pinhole aperture. Images were acquired at a resolution of 1024 × 1024‐pixel resolution, with a pixel size of approximately 1.55 μm × 1.55 μm (field of view: ~ 1.55 mm × 1.55 mm), using a 63 ×/1.20 NA Water Immersion objective (Leica Microsystems) at a scanning frequency of 400 Hz. Laser intensity, PMT gain, detector settings, AOTF (Acousto‐Optic Tunable Filters) attenuation and other imaging settings were kept constant across experimental groups to ensure comparability.

### Image processing and arrangement

Images were processed with consistent exposure times and gain settings across all series. Z‐stack maximum projections were generated and cropping or rotation was performed in imagej/fiji (Madison, WI, USA). For wide‐field images in Fig. S1C–F, deconvolution using the Iterative Deconvolve 3D and Diffraction PSF 3D plugins was applied to remove out‐of‐focus blur and enhance clarity. All figures, including flowcharts, were created and arranged using microsoft powerpoint (Microsoft Corporation, Redmond, WA, USA) (version 16.78) or adobe illustrator 2022 (San Jose, CA, USA) (version 26.4.1).

### Statistical analysis

Three sections per brain were analyzed from at least three individuals (with sample sizes indicated in the figure legends). Each brain section was treated as a single data point. All statistical analyses were performed and visualized using r. To determine the appropriate statistical test, data normality was assessed using the *Shapiro–Wilk* test with a significance level of α = 0.05. For normally distributed data, parametric tests were used: the *Welch t*‐test for comparisons between two samples, and the *Tukey HSD* test following a significant *ANOVA* (α = 0.05) for comparisons involving more than two groups. For non‐normally distributed data, the *Mann–Whitney U*‐test was used for unpaired two‐sample comparisons, and the *Dunn* test was applied following a significant *Kruskal–Wallis* rank sum test (α = 0.05) for multiple‐group comparisons.

## Author contributions

SR designed, supervised the experiments, analyzed the data, and cowrote the manuscript with JC, MT, and ND. JC, AH, MT, and TB conducted experiments and analyzed data. JC and MT analyzed and quantified the data. CV conducted the Hi‐C and 4C data analysis. All authors have reviewed and approved the final version of the manuscript for publication.

## Conflict of interest

The authors declare no conflict of interest.

## Supporting information


**Fig. S1.** Endogenous *mdka* expression and analysis of the *mdka* CRE3 enhancer in zebrafish embryos and adult telencephalon.
**Fig. S2.** Confocal imaging of *mdka* CRE transgene expression in type 1 radial glial cells (RGCs) in the adult zebrafish telencephalon under homeostatic conditions.
**Fig. S3.** Confocal analysis of *mdka* CRE transgene expression in type 2 radial glial cells (RGCs) in the injured telencephalon at 5 days post‐lesion (5 dpl).
**Table S1.** Coordinates of putative *mdka* CREs, the putative promoter, and known *mdka* CREs.
**Table S2.** Stable zebrafish lines used in this study.
**Table S3.** Primer oligonucleotides used for PCRs.
**Table S4.** Common plasmids used in this study.
**Table S5.** Primary and secondary antibodies used.

## Data Availability

The data that support the findings of this study are available in the main text and the Supporting Information of this article.
