# Supplementary material for: Multiple cis‐regulatory elements collaborate to control mdka expression in telencephalic neural stem cells of adult zebrafish during constitutive and regenerative neurogenesis
Source: FEBS J. 2025 Nov 19;293(7):1951–69. doi: 10.1111/febs.70345 (PMC13044984; doi:10.1111/febs.70345)
Supplement: Supplementary file 1 — Fig. S1. Endogenous mdka expression and analysis of the mdka CRE3 enhancer in zebrafish embryos and adult telencephalon. Fig. S2. Confocal imaging of mdka CRE transgene expression in type 1 radial glial cells (RGCs) in the adult zebrafish telencephalon under homeostatic conditions. Fig. S3. Confocal analysis of mdka CRE transgene expression in type 2 radial glial cells (RGCs) in the injured telencephalon at 5 days post‐lesion (5 dpl). Table S1. Coordinates of putative mdka CREs, the putative promoter, and known mdka CREs. Table S2. Stable zebrafish lines used in this study. Table S3. Primer oligonucleotides used for PCRs. Table S4. Common plasmids used in this study. Table S5. Primary and secondary antibodies used. [file FEBS-293-1951-s001.docx]

**Supporting Information**

**Multiple cis-regulatory elements collaborate to control *mdka* expression in telencephalic neural stem of adult zebrafish during constitutive and regenerative neurogenesis**

Jincan Chen, Masanari Takamiya, Agnes Hendriks, Tanja Beil, Csilla Varnai, Nicolas Diotel and Sepand Rastegar

**Supplementary Figures**


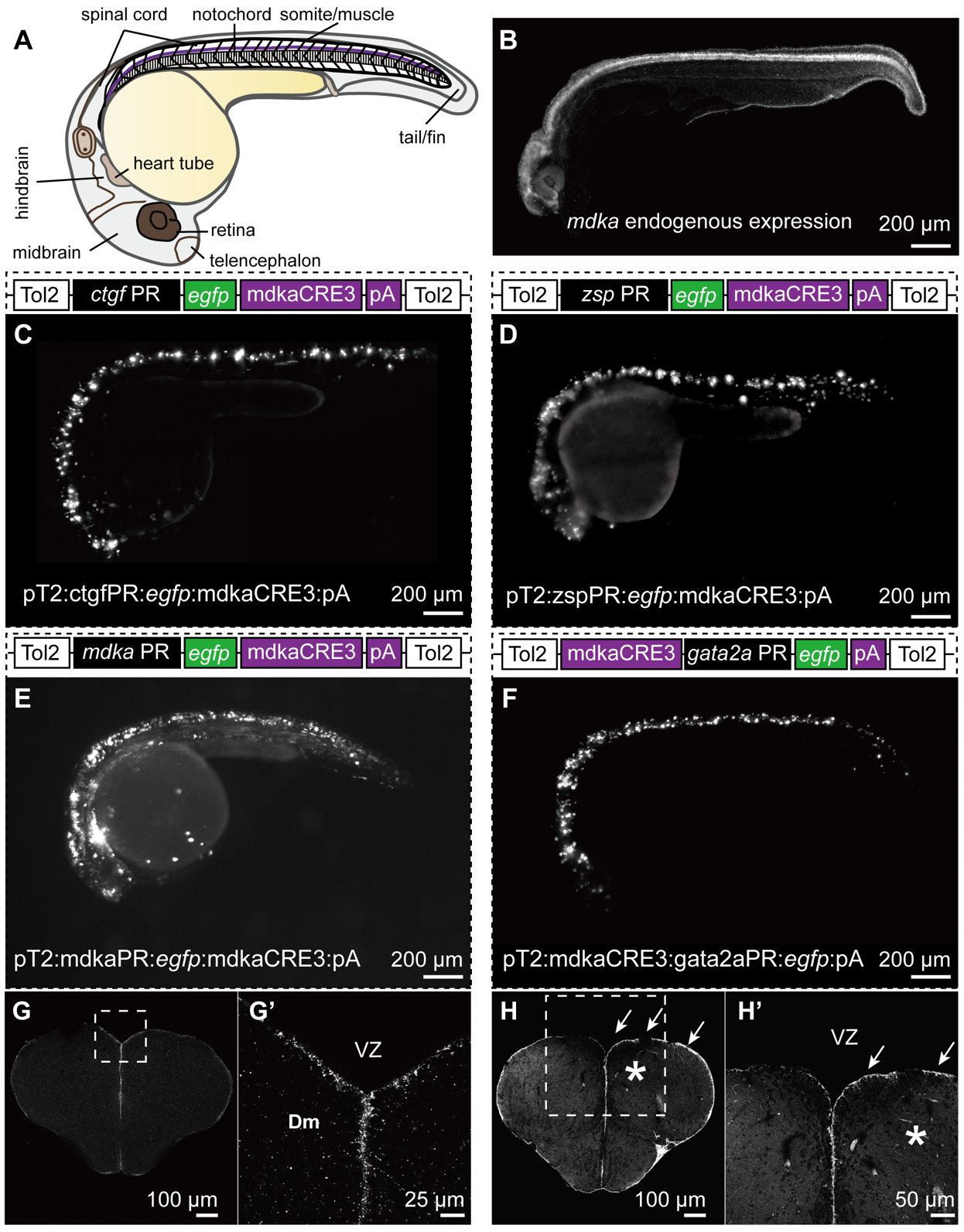


**Figure S1. Endogenous mdka expression and characterization of the mdka CRE3 enhancer in zebrafish embryos and adult telencephalon.**
**A,** Representative image of a 24-hours-post-fertilization (hpf) zebrafish embryo showing labeled anatomical structures, including the retina, heart tube, spinal cord, notochord, somites, and tail/fin fold. **B,** Fluorescent in situ hybridization (FISH) analysis of mdka expression in 24 hpf wild-type embryos shows detectable signals in the spinal cord, somites/muscles, fin/tail, and brain (including the telencephalon). **C–F,** Schematics and corresponding expression patterns of constructs used in transient transgenesis. Each construct contained the mdka CRE3 element paired with a different promoter: (**C**) ctgf promoter, (**D**) zsp promoter, (**E**) mdka promoter, and (**F**) minimal gata2a promoter. All constructs also included egfp and a polyA signal. The mdka promoter (mdkaPR, 500 bp) represents the intrinsic promoter of the mdka gene. Each construct drove consistent EGFP expression, primarily in the spinal cord and brain. All embryos are shown in lateral view with the anterior to the left. Although expression appeared punctate due to mosaicism inherent to transient assays, the spatial pattern of EGFP expression was consistent across promoter types, indicating that mdkaCRE3 activity is not promoter dependent. These data support the reproducibility of CRE3-driven expression and demonstrate that the observed patterns are attributable to the enhancer rather than promoter-specific effects. Representative embryos are shown; each construct was injected in three independent experiments with approximately 300 surviving embryos per experiment. About 70% of embryos consistently displayed the same EGFP expression pattern. **G–G′,** FISH analysis of uninjured wild-type telencephalon sections showing endogenous mdka expression along the ventricular zone (VZ), particularly in the dorsomedial pallium (Dm), lateral pallium (Dl), dorsal nucleus of the ventral telencephalon (Vd), and ventral nucleus of the ventral telencephalon (Vv). **G′** shows a higher-magnification view of the boxed VZ region in **G**. **H–H′,** FISH analysis of wild-type telencephalon sections at 5 days post-lesion (5 dpl) showing increased mdka expression in the injured hemisphere (arrows). **H′** shows a magnified view of the boxed VZ region in **H**. Representative images are shown; the same expression pattern in the adult zebrafish telencephalon was observed in at least 10 independent experiments. Scale bars as indicated; main images, 200 μm.


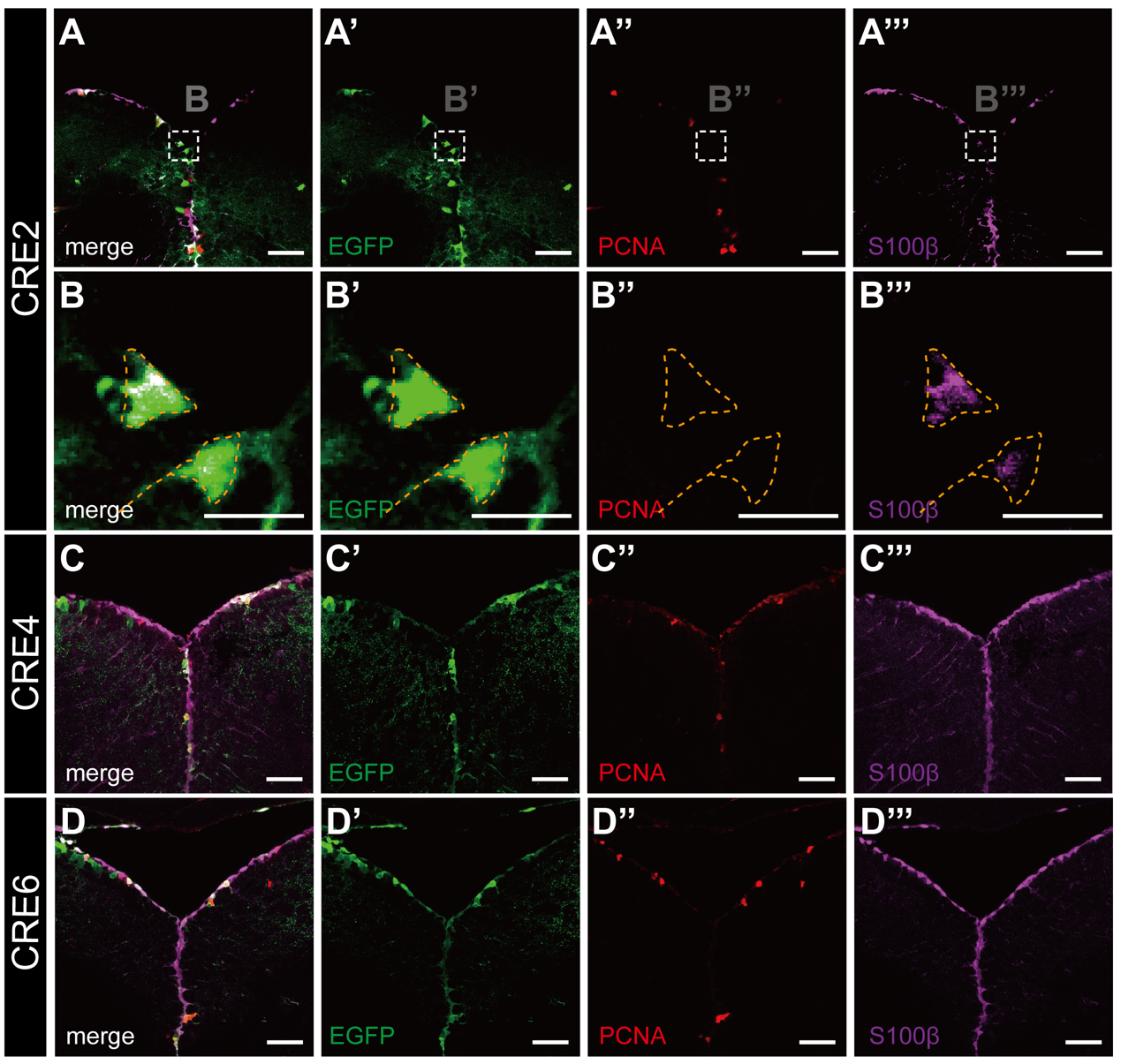


**Figure S2. Confocal analysis of mdka CRE transgene expression in type 1 radial glial cells (RGCs) in the adult zebrafish telencephalon under uninjured conditions.** (A–A’’’) Representative confocal z-projections showing EGFP⁺ cells co-immunostained for EGFP (green), PCNA (red), S100β (magenta), and merged channels (white) in the telencephalon of the CRE2 transgenic line under uninjured conditions. (B–B’’’) Higher-magnification images showing examples of type 1 RGCs in the uninjured telencephalon of the CRE2 line. Type 1 RGCs are defined as EGFP⁺/S100β⁺/PCNA⁻ cells. (C–C’’’) Representative confocal z-projections showing EGFP⁺ cells co-immunostained for EGFP (green), PCNA (red), S100β (magenta), and merged channels (white) in the telencephalon of the mdkaCRE4 transgenic line under uninjured conditions. (D–D’’’) Representative confocal z-projections showing EGFP⁺ cells co-immunostained for EGFP (green), PCNA (red), and S100β (magenta) in the telencephalon of the mdkaCRE6 line under uninjured conditions. These images correspond to the quantification shown in Figure 3N. Type 1 RGCs are defined as EGFP⁺/S100β⁺/PCNA⁻ cells, and type 2 RGCs as EGFP⁺/S100β⁺/PCNA⁺ cells. Scale bar: 25 μm.


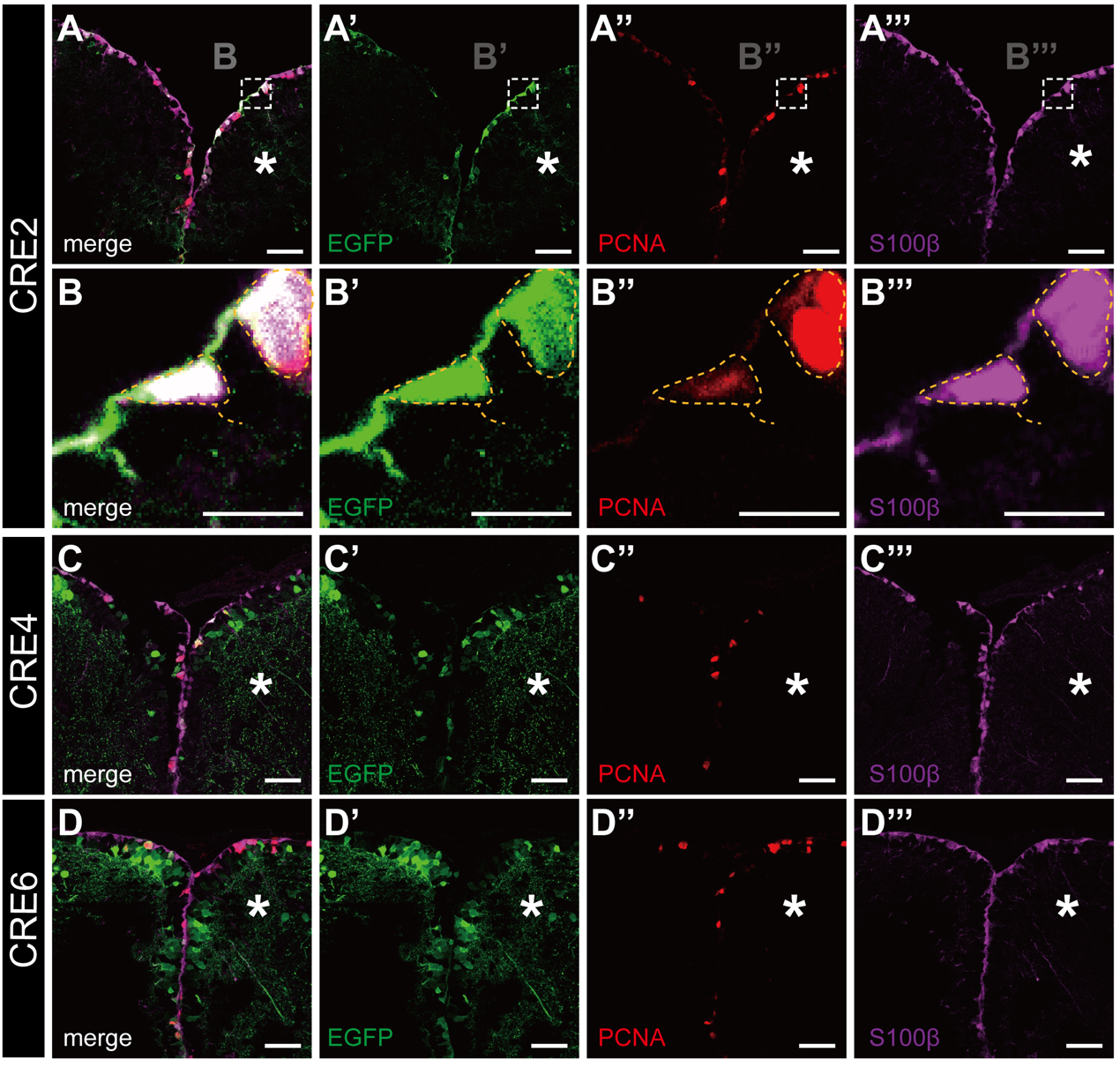


**Figure S3. Confocal analysis of mdka CRE transgene expression in type 2 radial glial cells (RGCs) in the injured telencephalon at 5 days post-lesion (5 dpl).** (A–A’’’) Representative confocal z-projections showing EGFP⁺ cells co-immunostained for EGFP (green), PCNA (red), S100β (magenta), and merged channels (white) in the telencephalon of the mdkaCRE4 transgenic line at 5 dpl. (B–B’’’) Higher-magnification images of the mdkaCRE2 transgenic line showing examples of type 2 RGCs (EGFP⁺/S100β⁺/PCNA⁺) in the injured hemisphere. (C–C’’’) Representative confocal z-projections showing EGFP⁺ cells co-immunostained for EGFP (green), PCNA (red), and S100β (magenta) in the telencephalon of the mdkaCRE4 line at 5 dpl. (D–D’’’) Representative confocal z-projections of the mdkaCRE6 transgenic line showing EGFP⁺ cells co-labeled for PCNA (red) and S100β (magenta) at 5 dpl. These images correspond to the quantification shown in Figure 4I. Type 1 RGCs are defined as EGFP⁺/S100β⁺/PCNA⁻ cells, and type 2 RGCs as EGFP⁺/S100β⁺/PCNA⁺ cells. Scale bars: 25 μm (A–A’’’, C–C’’’) and 10 μm (B–B’’’, D–D’’’).**Supplementary Tables**

**Table S1. Coordinates of putative *mdka* CREs, putative promoter and known *mdka* CREs.**

| Targets | GRCz10 coordinates | Size | Source |
| --- | --- | --- | --- |
| mdkaCRE1 | chr7:38877903-38878899 | 997 bp | This study |
| mdkaCRE2 | chr7:38889899-38891492 | 1594 bp | This study |
| mdkaCRE3 | chr7:38896921-38897894 | 974 bp | This study |
| mdkaCRE4 | chr7:38898055-38899236 | 1182 bp | This study |
| mdkaCRE5 | chr7:38900684-38903257 | 2574 bp | This study |
| mdkaCRE6 | chr7:38914234-38915726 | 1493 bp | This study |
| mdkaPR | chr7:38894821-38895320 | 500 bp | This study |
| mdka_e1 | chr7:38897699-38898662 | 964 bp | Weinberger et al. (2024) |
| mdka_e2 | chr7:38914567-38915876 | 1310 bp | Weinberger et al. (2024) |
| mdka_E20 | chr7:38914716-38916025 | 1310 bp | Thompson et al. (2020) |

**Table S2: Stable zebrafish lines used in this study.**

| Transgenic/mutant | Description | Source | Number of stable lines |
| --- | --- | --- | --- |
| *Tg(CRE1:gata2aPR:EGFP)* | CRE1 (997 bp) reporter line | This study | 4 |
| *Tg(CRE2:gata2aPR:EGFP)* | CRE2 (1594 bp) reporter line | This study | 4 |
| *Tg(CRE3:gata2aPR:EGFP)* | CRE3 (974 bp) reporter line | This study | 3 |
| *Tg(CRE4:gata2aPR:EGFP)* | CRE4 (1182 bp) reporter line | This study | 3 |
| *Tg(CRE5:gata2aPR:EGFP)* | CRE5 (2574 bp) reporter line | This study | 4 |
| *Tg(CRE6:gata2aPR:EGFP)* | CRE6 (1493 bp) reporter line | This study | 4 |
| *Tg(CRE2346:gata2aPR:EGFP)* | Reporter line that combines CRE2, CRE3, CRE4, and CRE6 | This study | 4 |
| WT ABO | Wildtype zebrafish | EZRC | - |
| WT LEO | Wildtype zebrafish | EZRC | - |

**Table S3. Primer oligos for PCR reaction mix.**

| **Target** | **Primer oligos** | **Anneal Temp.** |
| --- | --- | --- |
| CRE1 | Forward: tcttcactcgttgttactga;  Reverse: gtgatgctttctggattgaa | 49 °C |
| CRE2 | Forward: accttccgtcacttgtgcta;  Reverse: acgtgatcaggcccaactta | 55 °C |
| CRE3 | Forward: tcctgaagaacccaagaccc;  Reverse: taagcgcatgtgtgtgtgt | 54 °C |
| CRE4 | Forward: gtgtgtgtgtcatccagctg;  Reverse: gacattaaatgctcttctaaagccg | 52 °C |
| CRE5 | Forward: ccgctgggtaaaacatgtcc;  Reverse: ggtttgctgacattcgacgt | 55 °C |
| CRE6 | Forward: caatcaaagagtgcaaatctgc;  Reverse: gaggctgaaagtcacatgg | 51 °C |
| mdkaPR | Forward: gagagactcgagtttttcgcgcaacatgatta;  Reverse: gagagaggatcctgtcgaaagttccctcactg | 58 °C |

**Table S4: Common plasmids used in this study.**

| Plasmid | Size | Source/Details |
| --- | --- | --- |
| For standard cloning | | |
| pCR8/GW/TOPO | 2817bp | SpnR, from ThermoFisher Scientific |
| T2KHGpzGATA2C1 | 7311bp | destination vector, AmpR |
| pGEM-T Easy | 3016bp | AmpR, from Promega |
| For Gateway cloning | | |
| 228_p5E-MCS | 2810bp | 5’ entry clones, attL4-R1, KanR, insert with multiple-cloning site from pBluescript |
| 237_pME-MCS | 2765bp | middle entry clones, attL1-L2, KanR, insert with multiple-cloning site from pBluescript |
| 383_pME-EGFP | 3327bp | middle entry clones, attL1-L2, KanR, insert with EGFP |
| 302_p3E-polyA | 2838bp | 3’ entry clones, attR2-L3, KanR, insert with SV40 late polyA signal |
| 392_pDestTol2pA | 7903bp | destination vectors, attR4-R3, AmpR or ChlorR, grow in ccdB tolerant cells, insert with attR4-R3 gate with SV40 polyA flanked by Tol2 inverts |

**Table S5: List of primary and secondary antibodies.**

| **Antibodies** | **Name** | **Dilution** | **Source** |
| --- | --- | --- | --- |
| Primary | chicken anti-EGFP | 1:1000 | Aves labs, Davis, USA |
|  | mouse anti-PCNA | 1:500 | Agilent, Santa Clara, USA |
|  | rabbit anti-S100β | 1:2 | Agilent, Santa Clara, USA |
|  | DAPI | 1:2000 | Agilent, Santa Clara, USA |
| Secondary | AlexaFluor 488 anti-chicken | 1:1000 | Invitrogen, Carlsbad, USA |
|  | AlexaFluor 546 anti-mouse | 1:1000 | Invitrogen, Carlsbad, USA |
|  | AlexaFluor 633 anti-rabbit | 1:500 | Invitrogen, Carlsbad, USA |
